# Phase intensity nanoscope (PINE) opens long-time investigation windows of living matter

**DOI:** 10.1038/s41467-023-39624-w

**Published:** 2023-07-18

**Authors:** Guangjie Cui, Yunbo Liu, Di Zu, Xintao Zhao, Zhijia Zhang, Do Young Kim, Pramith Senaratne, Aaron Fox, David Sept, Younggeun Park, Somin Eunice Lee

**Affiliations:** 1 Department of Electrical & Computer Engineering, Biomedical Engineering, Applied Physics, Biointerfaces Institute, Macromolecular Science & Engineering, University of Michigan, Ann Arbor, MI USA; 2 Department of Biomedical Engineering, University of Michigan, Ann Arbor, MI USA; 3 Department of Mechanical Engineering, University of Michigan, Ann Arbor, MI USA

**Keywords:** Imaging and sensing, Super-resolution microscopy

## Abstract

Fundamental to all living organisms and living soft matter are emergent processes in which the reorganization of individual constituents at the nanoscale drives group-level movements and shape changes at the macroscale over time. However, light-induced degradation of fluorophores, photobleaching, is a significant problem in extended bioimaging in life science. Here, we report opening a long-time investigation window by nonbleaching phase intensity nanoscope: PINE. We accomplish phase-intensity separation such that nanoprobe distributions are distinguished by an integrated phase-intensity multilayer thin film (polyvinyl alcohol/liquid crystal). We overcame a physical limit to resolve sub-10 nm cellular architectures, and achieve the first dynamic imaging of nanoscopic reorganization over 250 h using PINE. We discover nanoscopic rearrangements synchronized with the emergence of group-level movements and shape changes at the macroscale according to a set of interaction rules with importance in cellular and soft matter reorganization, self-organization, and pattern formation.

## Abstract (web summary)

The authors present phase intensity nanoscope (PINE), which uses an integrated phase-intensity multilayer thin film to localize randomly distributed nanoprobes and resolve sub-10 nm cellular architectures. They demonstrate dynamic imaging of nanoscopic reorganization over 250 h and find that nanoscale rearrangements emerging into macroscale rearrangements are synchronized.

## Introduction

How macroscale groups emerge over time from their individual constituents is fundamental to self-assembly, self-organization, and pattern formation in material science^1^ and biology^2,3^. The diverse range of emergent dynamics^4–6^ spans at least three orders of magnitude in length scales and at least five orders of magnitude in time scales. Excellent prior arts of fluorescence super-resolution^7–11^—in which STED, MINSTED, MINFLUX intentionally apply photobleaching and STORM, dSTORM, PALM, dPALM cycle fluorescence states before photobleaching—have unlocked optical imaging across the spatial domain down to the sub-10 nm length scale. However, light-induced degradation of fluorophores is permanent which ultimately sets an irreversible photobleaching limit and restricts observation time, making long-time super-resolution difficult to achieve (Fig. S1). Thus, a long-time super-resolution method that can open a long-time investigation window down to the sub-10 nm length scale is needed for visualizing emergent nanoscale-to-macroscale dynamics but has not been yet reported.

Nanoprobes undergoing elastic scattering processes without photobleaching offers a nonbleaching approach to achieve long-time super-resolution. However, current methods^12–16^ by moving analyzers/polarizers are subject to displacements and imprecise localization, making precise super-resolution difficult. Current demonstrations (Fig. S2) have resolved only two to few nanoprobes, whereas populations (thousands) are needed to form patterns of underlying cellular architectures. In addition, all of the aforementioned methods have not achieved super-resolution down to the sub-10 nm length scale.

**Fig. 1:**
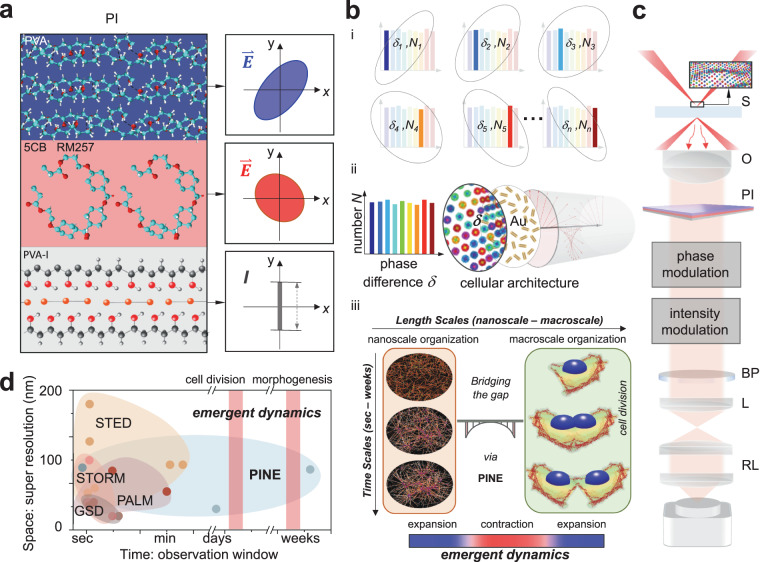
Principle of the nonbleaching phase-intensity nanoscope, PINE, for living matter. **a** Phase-intensity PI: Integrated phase-intensity multilayer thin film consisting of polyvinyl alcohol/liquid crystalline polymers, enable precise control of phase differences between electric field components. Scattered light is reshaped according to phase modulation. Phase modulation is then converted to intensity modulation such that the resulting variation of intensity corresponds to subsets of nanoprobes labeling cellular architectures. **b** Concept of PINE: (i) PI precisely modulates phase differences *δ*_*n*_ corresponding to subsets of nanoprobes *N*_*n*_ within the population. *N*: number of nanoprobes. *δ*: phase difference between electric field components. (ii) Randomly distributed nanoprobes (Au nanorods) form patterns of the underlying cellular architectures. Using PI, nanoprobes exhibit phase differences between electric field components in a stochastic manner. (iii) PINE opens a long-time investigation window to investigate emergent nanoscale-to-macroscale dynamics: in cell division, reorganization of individual constituents at the nanoscale emerges into group-level movements and shape changes at the macroscale over time. **c** Set-up. Darkfield configuration illuminates a nanoprobe-labeled sample (S) in a temperature- and gas-controlled flow chamber. The collected scattered light by objective (O) is phase-intensity modulated (PI) and bandpass filtered (BP). To increase the system’s magnification, relay lenses (RL) were added to increase the effective focal length of the tube lens (L). After phase-intensity separation, the resulting intensity variation corresponds to subsets of nanoprobes. **d** Fluorescence super-resolution methods, such as ground state depletion (GSD), stimulated emission depletion (STED), photo-activated localization microscopy (PALM), and stochastic optical reconstruction microscopy (STORM), have pushed spatial resolution beyond the diffraction limit (y-axis) (full table in supporting information Fig. S1). PINE creates new nanoscopic opportunities along the time axis (x-axis) for investigations demanding long-time observation windows.

Herein, we present a nonbleaching phase-intensity nanoscope, PINE, to open a long-time investigation window of living matter down to the sub-10 nm length scale. We designed an integrated phase-intensity multilayer thin film of polyvinyl alcohol and liquid crystalline polymers (herein referred to as PI) (Fig. 1a), enabling populations of randomly distributed nanoprobes (gold nanorods) to exhibit phase differences between electric field components in a stochastic manner (Fig. 1b, c). Owing to the tunability of PI, we achieved phase-intensity separation of nanoprobes within a diffraction-limited region by modulating phase differences between electric field components. As PI is displacement-free, precise localization of nanoprobe populations (thousands) form patterns of underlying cellular architectures enabling precise super-resolution down to the sub-10 nm length scale. We quantified distributional parameters to overcome a physical limit to resolve sub-10 nm cellular architectures. Using nonbleaching PINE, we obtained the dynamic imaging of nanoscale reorganization over 250 h, which outperforms state-of-the-art fluorescence super-resolution by more than two orders of magnitude. With a long-time investigation window by PINE (Fig. 1d), we identified emergent synchronized individual-group contraction-expansion, SYNC, in which nanoscopic rearrangements coordinated with the emergence of group-level movements and shape changes at the macroscale during the cell division process according to a set of interaction rules. We are able to achieve long-time sub-10 nm nanoscopy and follow the emergence of large scale collectives involving hundreds of cellular architectures (>900) in cells, all of which cannot be obtained using existing fluorescence^7–11^ or nonbleaching^12–16^ methods.

## Results

### Principle of PI device design

**Fig. 2:**
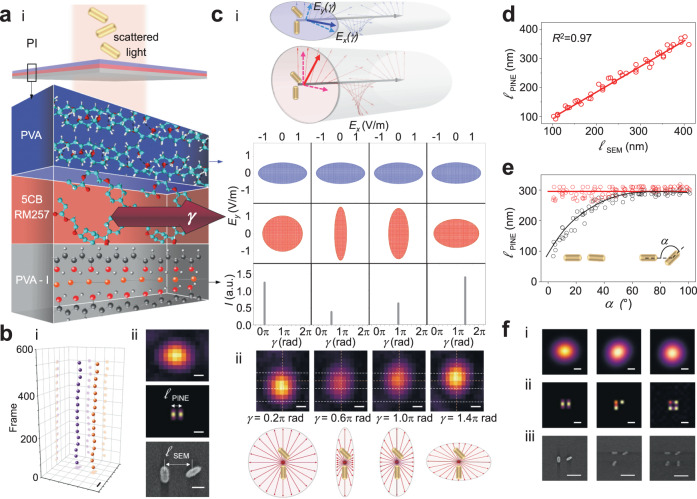
PI phase-intensity separates multiple nanoprobes within a diffraction-limited region by distinguishing phase differences between electric field components. **a** PI: Integrated phase-intensity multilayer stack of polyvinyl alcohol, liquid crystal and liquid crystal polymer, and polyvinyl alcohol-iodine layers. **b** Phase-intensity separation: (i) Scatter plot: Images repeatedly acquired over the modulation range *γ* from 0 to 2π rad, generating image stack of ~500 total frames. The spatial positions of each nanorod were determined by taking the median value. Scale bar: 100 nm. (ii) Diffraction-limited darkfield image. Scale bar: 200 nm. PINE-resolved image. Color represents intensity after phase-to-intensity conversion. Scale bar: 200 nm. Scanning electron microscopy image. Scale bar: 100 nm. **c** (i) Schematic of multiple nanoprobes (gold nanorods) within a diffraction-limited region: Reshaped scattered light exhibited phase difference between electric field components *E*_*x*_ and *E*_*y*_ to distinguish multiple nanoprobes within a diffraction-limited region. Calculated electric field and intensity amplitudes: Scattered light was reshaped (top row), phase modulated (middle row), and intensity modulated (bottom row). (ii) Experimental darkfield images at *γ* = 0.2π, 0.6π, 1.0π, and 1.4π rad acquired by PI. Scale bar: 200 nm. Schematic of phase modulation by PI. **d** Benchmarking of localization: Comparison of ${\ell }_{{{{{{\rm{PINE}}}}}}}$ imaged by PINE and ${\ell }_{{{{{{\rm{SEM}}}}}}}$ measured by scanning electron microscopy. i-1 through i-5 correspond to Fig. S10. **e** Localization precision: Red curve ${\ell }_{{{{{{\rm{PINE}}}}}}}$: Displacement-free PINE showing precise localization as $\alpha \to 0$. Black curve ${\ell }_{{{{{{\rm{control}}}}}}}$: Displacement control (Supplemental Note I) showing increasingly imprecise localization as $\alpha \to 0$. **f** (i) Diffraction-limited darkfield images of multiple nanoprobes [two (left), three (middle), four (right)]. Scale bar: Scale bar: 200 nm. (ii) PINE-resolved images of multiple nanoprobes [two (left), three (middle), four (right)]. Color represents intensity after phase-to-intensity conversion. Scale bar: 200 nm. (iii) Scanning electron microscopy images of multiple nanoprobes [two (left), three (middle), four (right)]. Scale bar: 250 nm.

We fabricated PI (Fig. 2a) for precise control of phase differences between electric field components to accomplish phase-intensity separation of nanoprobes within a diffraction-limited region (Fig. 2b). The compact multilayer design (~600 µm thickness; Fig. S3) was integrated into the infinity space between the objective and tube lens (Fig. 1c), enabling high transmission for nanoscopy. Scattering^17–23^ without photobleaching was used. Input light to PI was the scattered light from a population of nanoprobes (gold nanorods). Scattered light was reshaped, phase modulated, and converted from phase to intensity by PI. Phase differences between electric field components enable nanoprobes within a diffraction-limited region to be distinguished from one another by phase-intensity separation. Because the optical axis is stationary, PI is displacement-free for precise nanoscopy. Thus,

1 $${{{{{\bf{E}}}}}}=\,\frac{1}{2}\left[\begin{array}{cc}1 & 0\\ 0 & 0\end{array}\right]\left[\begin{array}{cc}{e}^{i\frac{\gamma }{2}}+{e}^{-i\frac{\gamma }{2}}\, & {e}^{i\frac{\gamma }{2}}-{e}^{-i\frac{\gamma }{2}}\\ {e}^{i\frac{\gamma }{2}}+{e}^{-i\frac{\gamma }{2}} & {e}^{i\frac{\gamma }{2}}-{e}^{-i\frac{\gamma }{2}}\,\end{array}\right]\left[\begin{array}{cc}\,{e}^{i{\delta }_{x}} & 0\\ 0 & {e}^{i{\delta }_{y}}\end{array}\right]\left[\begin{array}{c}{E}_{x}\\ {\begin{array}{c}E\end{array}}_{y}\end{array}\right]$$

where $\delta={\delta }_{y}-{\delta }_{x}$ is the phase difference between the scattered electric field components *E*_*x*_ and *E*_*y*_, $\gamma={\int }_{0}^{D}\frac{{n}_{e}{n}_{o}{dz}}{\sqrt{{n}_{e}^{2}{{{cos }}}^{2}\theta+{n}_{o}^{2}{{{sin }}}^{2}\theta }}\,-{n}_{o}D$ is the phase retardation, and *D*, *n*_*o*_, *n*_*e*_, and *θ* are the layer thickness, ordinary refractive index, extraordinary refractive index, and molecular alignment with respect to the applied electric field. The Jones matrices represent a polyvinyl alcohol layer designed as a fixed retarding element for reshaping, 4-cyano-4’-pentylbiphenyl and 4-(3-acryloyoxypropyloxy) benzoic acid 2-methyl-1,4-phenylene ester layer designed as a variable retarding element for phase modulation, and polyvinyl alcohol-iodine layer designed as a fixed linear polarizing element for phase-to-intensity conversion. The resulting variation of intensity corresponded to subsets of nanoprobes where

2 $$I\left(\gamma \right)={{{cos }}}^{2}\left(\frac{\gamma }{2}\right){E}_{x}^{2}+{{{sin }}}^{2}\left(\frac{\gamma }{2}\right){E}_{y}^{2}+2{{cos }}\left(\frac{\gamma }{2}\right){E}_{x}{{sin }}\left(\frac{\gamma }{2}\right){E}_{y}{{sin }}(\delta )$$

PI was fabricated by a deposition process, where precursor of 4-(3-acryloyoxypropyloxy) benzoic acid 2-methyl-1,4-phenylene ester, 1-hydroxycyclohexyl phenyl ketone, and 4-cyano-4′-pentylbiphenyl was deposited and cured between two indium tin oxide-polyethylene terephthalate substrates on top of a polyvinyl alcohol-iodine layer. Polyvinyl alcohol was layered on top of the aforementioned layers. We characterized the material alignment uniformity (Fig. S4) and millisecond response time (Fig. S5). During phase-intensity modulation, the center coordinates were expressed as

$${x}_{\gamma }={x}_{0},$$

3 $${y}_{\gamma }={y}_{0}$$

where *x*_0_ and *y*_0_ are the center coordinates prior to modulation. Here, the optical axis was stationary such that (*x*_*γ*_, *y*_*γ*_) is equal to (*x*_0_, *y*_0_), enabling zero displacement for precise nanoscopy. This allows for precise localization and eliminates potential error propagation in scaling up^24^. In scaling up, populations (thousands) of precisely localized nanoprobes form patterns of underlying architectures of arbitrary shape. Conventional microscopy blurs the distribution of nanoprobes convolved with the point spread function (PSF) of the imaging system. To obtain sub-10 nm information, nanoprobes within a diffraction-limited region were isolated by phase-intensity separation with zero displacement for precise nanoscopy such that the distribution of precisely localized nanoprobes forms patterns of underlying architectures. From the distribution of precisely localized nanoprobes, surface or curvilinear features ${{{{{\bf{f}}}}}}(p)$ were defined, in which the Euclidean distance from nanoprobe positions to their projection ${{{{{\bf{f}}}}}}(p)$ was minimized. By defining a new parameter *χ*, sub-10 nm information was obtained from the distribution *χ*(*p*):

4 $$\chi \left(p\right)=\frac{1}{n}{\left|\left|\left({Pk}{x}_{i},{Pk}{y}_{i}\right)-{{{{{\bf{f}}}}}}\left(p\right)\right|\right|}_{2}$$

where $({Pk}{x}_{i},{Pk}{y}_{i})$ represents the distribution of precisely localized nanoprobes (see “Methods” section for details).

### Sub-10 nm PINE

To validate the phase-intensity separation concept, we investigated nanoprobes (gold nanorods) fabricated by electron beam lithography in order to systematically vary geometrical parameters (Figs. S6, S7a, S8a). For this validation study, we chose electron beam lithography over chemical synthesis because of precise fabrication control of geometrical parameters. As a model, we fabricated plasmonic gold nanorods to determine whether phase differences between electric field components could be used to segregate nanorods in a diffraction-limited region. Due to their high aspect ratio geometries significantly smaller than the wavelength, conduction electrons collectively oscillate in phase, resulting in strong scattering cross-sections^25–44^. As an initial test, the scattered light from a pair of nanorods was reshaped by PI. When PI modulated *γ* from 0 to 2π rad, the phase difference between *E*_*x*_ and *E*_*y*_ was varied from −π το π rad and the positions of each nanorod emerged. PINE precisely determined the spatial positions of each gold nanorod (Fig. 2b and c). Localization was firstly optimized based on a geometrical parameter, aspect ratio, via simulation (Fig. S9), whereby we then selected aspect ratio 2.9 to be fabricated by electron beam lithography. Due to the delicate nature of electron beam fabricated samples, a long working distance, low NA objective and dry condenser with a diffraction limit of 450 nm were used in Fig. 2. In Fig. 2d, we benchmarked geometrical parameter $\ell$ imaged by PINE against $\ell$ measured by scanning electron microscopy. We fixed *α* and then varied $\ell$ between 100 nm to 400 nm. We validated localization accuracy in agreement with scanning electron microscopy measurements with an *R*^2^ of 0.97 (Figs. 2d, S10). We then turned to geometrical parameter *α*. In Fig. 2e, we varied *α* between 2° to 100° at a fixed known subdiffraction $\ell$ of 300 nm which was below the diffraction limit of 450 nm. Owing to the displacement-free multilayer (Supplemental Note I; Figs. S11, S12), nearly identical nanorods as $\alpha \to 0$ could be precisely localized (red curve Fig. 2e). The minimum *α* that two proximal nanoprobes will require at an effective subdiffraction $\ell$ is 2°. To answer the question of the minimum *α* that two nanoprobes at the minimum $\ell$ will require, we then varied both *α* and $\ell$. As both *α* and $\ell$ were varied, different effective resolutions were observed. Figure S13 shows that the minimum *α* that two nanoprobes at the minimum $\ell$ of 80 nm is 45°. Smaller *α* are resolvable at larger subdiffraction $\ell$ below the diffraction limit. We verified multiple nanoprobes within a diffraction-limited region were phase-intensity separated by PI (Fig. 2f, Figs. S7, S8). We estimate approximately twenty nanorods can be resolved in a diffraction-limited spot.

**Fig. 3:**
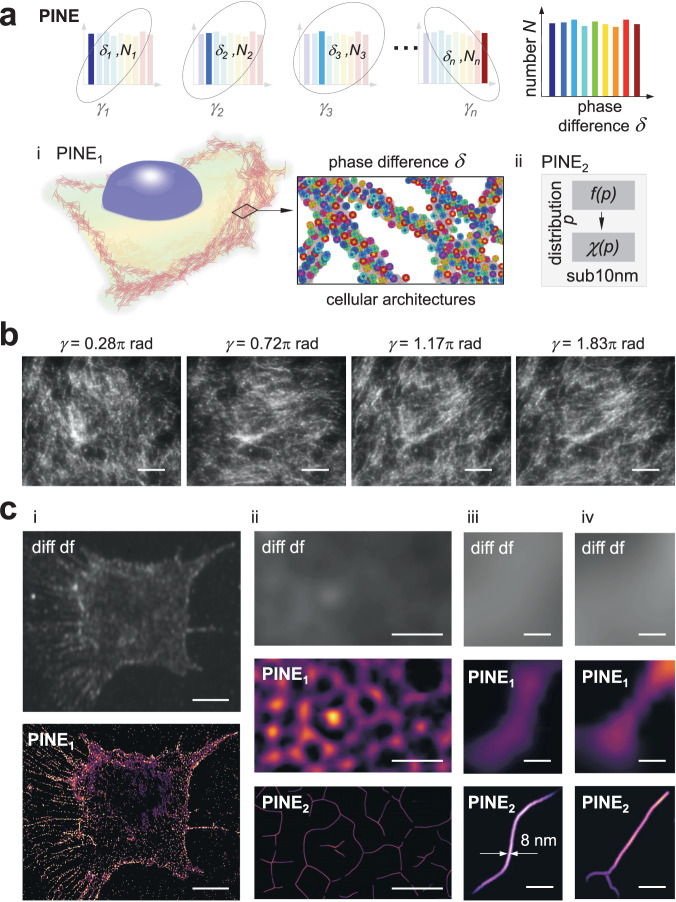
PINE reveals sub-10 nm cellular architectures in SH-SY5Y cells. **a** PINE: (i) PINE_1_: PI precisely modulates phase differences *δ*_*n*_ corresponding to subsets of nanoprobes *N*_*n*_ within the population. *N*: number of nanoprobes (gold nanorods). *δ*: phase difference between electric field components. In cells, randomly distributed nanoprobes (gold nanorods) form patterns of the underlying cellular architectures. Using PI, nanoprobes exhibit phase differences between electric field components in a stochastic manner. (ii) PINE_2_: Surface or curvilinear features ${{{{{\boldsymbol{f}}}}}}(p)$ were defined. Parameter *χ* was obtained from the pattern of precisely localized nanoprobe populations and ${{{{{\boldsymbol{f}}}}}}(p)$. From the distribution *χ*(*p*), sub-10 nm information was obtained. **b** Experimental darkfield images of cellular architectures (actin) at *γ* = 0.28π, 0.72π, 1.17π, and 1.83π rad acquired using PI. Scale bar: 20 µm. **c** (i) (top) Diffraction-limited darkfield image of SH-SY5Y cell. (bottom) PINE_1_-resolved image of SH-SY5Y cell. Scale bar: 10 μm. (ii) (top) Diffraction-limited darkfield image. (middle) PINE_1_-resolved image. (bottom) PINE_2_-resolved image. Scale bar: 500 nm. (iii) PINE revealed sub-10 nm actin filament architecture in SH-SY5Y cells: (top) Diffraction-limited darkfield image. (middle) PINE_1_-resolved image. (bottom) PINE_2_-resolved image. Scale bar: 80 nm. (iv) PINE revealed sub-10 nm branched actin architecture in SH-SY5Y cells: (top) Diffraction-limited darkfield image. (middle) PINE_1_-resolved image. (bottom) PINE_2_-resolved image. Scale bar: 80 nm.

Randomly distributed nanoprobes introduce a stochastic variation within a population (Fig. 3a). We reasoned their phase differences between electric field components should be also stochastically distributed. We utilized this stochasticity for the purpose of enhancing resolution. In order to form patterns of underlying cellular architectures, structurally stabilized nanoprobes in which scaling up to distributions of nanoprobes are required. Traditionally, gold nanorods^45,46^ are synthesized with a strong cationic charge from hexadecyltrimethylammonium bromide capping in which achieving 100% CTA+ free necessary for structural stability has been historically difficult due to the strong cationic charge^47^. Here, we chemically synthesized with bromide-free surfactants (Supplementary: sample preparation section) to yield 100% CTA+ free monodisperse gold nanorods (material composition characterization: Fig. S14) with size variability of ~5%^48,49^. We achieved structurally stabilized distributions of nanoprobes for nanoscopic imaging (Fig. 3). In scaling up, populations of precisely localized nanoprobes formed patterns of the underlying cellular architectures. In cells, actin plays key roles both in bundled and single filament forms. Actin was labeled with CTA+ free nanorods conjugated with antibodies targeting actin. Scanning electron microscopy images verified nanoprobes were randomly distributed along actin bundles (Fig. S15). PI reshaped scattered light from the randomly distributed population of nanoprobes. PI then modulated *γ* from 0 to 2π rad (Fig. 3b) which varied the phase difference between *E*_*x*_ and *E*_*y*_ from –π το π rad. After phase-to-intensity conversion, the resulting variation of intensity corresponded to subsets of precisely localized nanoprobes (PINE_1_: see “Methods” section for details). To form patterns, we were able to scalably resolve nanoprobe populations from few (Figs. 2, S6–S8) to 7769 (Figure S16) nanoprobes (up to ~10^2^/µm^2^). Scaling up allowed for distributions of precisely localized nanoprobes to form patterns of the underlying actin bundles (Fig. S17, Supplemental Note II) in agreement with scanning electron microscopy (Fig. S18). Underlying architectures were identified for nanoprobe labeling densities of ~10^2^/µm^2^ based on distribution of distances from every nanoprobe to its first neighbor following a distribution function^50^ (Fig. S19), enabling continuous features to be defined from the discrete nanoprobe distributions in agreement with scanning electron microscopy (Fig. S18).

In contrast to bundles we observed earlier, actin is often in single filament form in cells^51^. In SH-SY5Y cells, nanoprobes within a diffraction-limited region were isolated by phase-intensity separation by modulating *γ* from 0 to 2π rad using PI. Precisely localized nanoprobes formed patterns of the underlying single filaments. While the minimum $\ell$ is 80 nm (Fig. S13), sub-10 nm information can be obtained. Surface or curvilinear features ${{{{{\bf{f}}}}}}(p)$ were defined. Parameter *χ* was obtained from the pattern of precisely localized nanoprobe populations and ${{{{{\bf{f}}}}}}(p)$. From the distribution *χ*(*p*) (PINE_2_: see “Methods” section for details), PINE revealed actin width to be 8.0 nm (Figs. 3c, S20) consistent with width of actin filaments in cells^52^. Notably, sub-10 nm features revealed single filament architectures (Fig. 3c iii) and branched architectures (Fig. 3c iv) which were indistinguishable from each other in the absence of sub-10 nm information. Both controls—absence of both nanoprobes and antibody; presence of nanoprobes without antibody—were negative (Fig. S21). To our knowledge, this is the first sub-10 nm demonstration by a nonbleaching nanoscopy method owing to the displacement-free mechanism of PI for precise nanoscopy.

### Long-time PINE (Model: emergent dynamics SYNC)

A longstanding question is how macroscale groups emerge from their individual constituents. Commonalities between diverse systems suggest general rules^53–56^ guiding the collective reorganization of individuals into groups. This has prompted models of emergent phenomena ranging from microscale forces^57–59^ and cellular forces at the macroscale^60,61^ to microscale chemical reactions^62,63^ and macroscale reactions among networks^64^, underscoring a need for experiments and theories bridging across diverse length scales and time scales. Using PINE, the ability to bridge across diverse length scales and time scales provides a unique opportunity to shed light on the collective reorganization of individuals and groups (Fig. 1b iii).

**Fig. 4:**
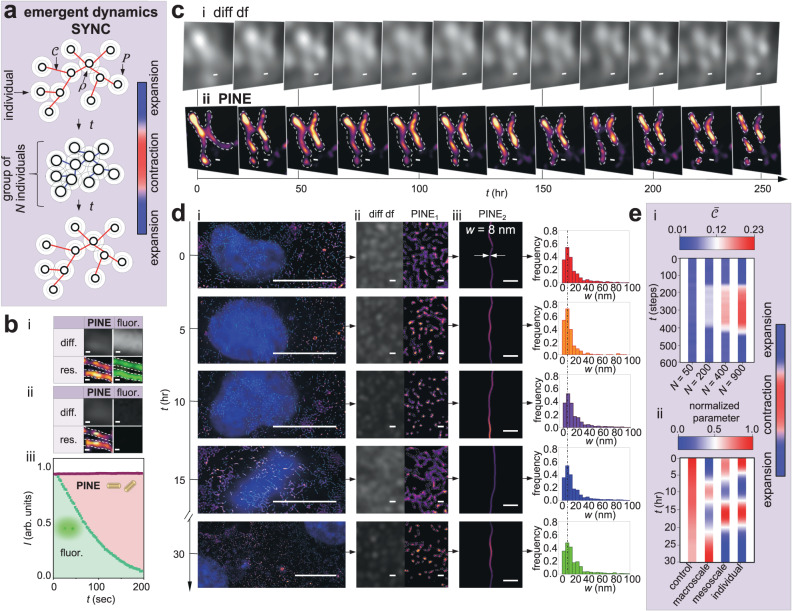
Synchronized individual-group contraction-expansion, SYNC, emerged during the cell division process. **a** SYNC Model: Conceptual schematic of SYNC model following Eqs. 5–7 where *t* = time, *Ρ* = long-range space, *ρ* = short-range space, $\left(1-{{{{{\mathcal{C}}}}}}\right)$ = connection. **b** Benchmarking of nonbleaching nanoscopy: fluorescence super-resolution versus PINE. (i) Time *t* = 0 s: Diffraction-limited darkfield image (top left) and PINE-resolved image (bottom left) at time *t* = 0 s. Diffraction-limited fluorescence image (top right) and fluorescence super-resolution resolved image (bottom right) at time *t* = 0 s. Scale bar: 200 nm. (ii) Time *t* = 200 s: Diffraction-limited darkfield image (top left) and PINE-resolved image (bottom left) at time *t* = 200 s. Diffraction-limited fluorescence image (top right) and fluorescence super-resolution resolved image (bottom right) at time *t* = 200 s. Scale bar: 200 nm. (iii) Graph of average scattering intensity over time for fluorescence super-resolution versus PINE. PINE (purple), fluorescence super-resolution (green). **c** Validation of long-time PINE: Diffraction-limited darkfield and PINE-resolved time course of actin dynamics. Scale bar: 150 nm. **d** (i) Cell division at the macro-, meso- and individual levels: PINE_1_-resolved macroscale time course in SH-SY5Y cells (left) overlaid with fluorescence (nucleus). Scale bar: 10 μm. (ii) Diffraction-limited darkfield images and PINE_1_-resolved time course of mesoscale group of individual filaments in SH-SY5Y cells (middle). Scale bar: 500 nm. (iii) PINE_2_-resolved time course of individual filaments in SH-SY5Y cells (right). Scale bar: 80 nm. Time course histograms of resolved actin filament width by PINE. **e** SYNC: (i) Theory: Model applied to cell division. Output connection $\left(1-{{{{{\mathcal{C}}}}}}\right)$ over time for different group sizes of *N* individuals according to Eqs. 5–7. $\bar{{{{{{\mathcal{C}}}}}}}$: mean ${{{{{\mathcal{C}}}}}}$ for a group size of *N* individuals, *c* = 0.5 and 5, *Ρ* = 5 μm, *ρ* = 500 nm. (ii) Experiment: Synchronized expansion-contraction at the macro-, meso- and individual levels. Time course contraction-expansion graph was normalized in order to plot together undivided control (unnormalized Fig. S25), macroscale (unnormalized Fig. S26), mesoscale (unnormalized Fig. S28), and individual (unnormalized Fig. S27). Number of individuals *N* = 904.

When large scale collectives involve hundreds of individual constituents, increasing experimental evidence^5,65^ suggests that collective behavior may arise from local interactions versus global instructions. From a set of local interaction rules, we describe synchronized individual-group contraction-expansion, SYNC, in which individual constituents coordinate with group level movements and shape changes at the macroscale (Fig. 4a). A group of *N* individuals will expand or contract based on connectivity between neighboring individuals. Connectivity *c* is defined as the ability of individuals to connect with one or more others. Rule 1: An individual will occupy more short-range space to increase connectivity with neighbors.

5 $${s}_{i}=\mathop{\sum}\limits_{k\ne i}\frac{{p}_{i}\left(t\right)-{p}_{k}\left(t\right)}{\left|{p}_{i}\left(t\right)-{p}_{k}\left(t\right)\right|}$$

where *s*_*i*_ is the direction of movement of individual *i* within a short-range space *ρ*, *p*_*i*_ is the position vector of an individual *i*, and *p*_*k*_ is the position vector of a neighbor *k* within the short-range space *ρ*. This simulates the movement direction of an individual to alter connectivity with short-range neighbors. Rule 2: Long-range space between individuals will decrease to increase connectivity with neighbors.

6 $${l}_{i}=-\mathop{\sum}\limits_{j\ne i}\frac{{p}_{i}\left(t\right)-{p}_{j}\left(t\right)}{\left|{p}_{i}\left(t\right)-{p}_{j}\left(t\right)\right|}$$

where *l*_*i*_ is the direction of movement of individual *i* within a long-range space *Ρ*, *p*_*i*_ is the position vector of an individual *i*, and *p*_*j*_ is the position vector of a neighbor *j* within a long-range space *Ρ*. This represents the movement direction of an individual to alter connectivity with long-range neighbors. Rule 3: Group contracts with increased connectivity. Group expands with decreased connectivity.

7 $${M}_{i}\left(t+\Delta t\right)=-\mathop{\sum}\limits_{i\ne j}\frac{c[{p}_{i}\left(t\right)-{p}_{j}\left(t\right)]-{s}_{i}}{\left|c[{p}_{i}\left(t\right)-{p}_{j}\left(t\right)]-{s}_{i}\right|}$$

where *M*_*i*_ is the direction of movement of individual *i* over time *t* influenced by connectivity *c*. This manifests in expansion or contraction of the group on the basis of connectivity between neighboring individuals. Thus, *c* was increased for expansion, and *c* was decreased for contraction. SYNC was quantified by resultant connection defined as $\left(1-{{{{{\mathcal{C}}}}}}\right)$. We found there was a threshold group size for collective behavior at the group level. At the threshold group size, expansion-contraction behavior commenced (Fig. 4e i), meaning sufficient interactions between individuals and neighboring individuals were necessary. Thereafter, as the group size became larger, the expansion-contraction behavior increased. Threshold group size may impact expansile and contractile forces of a connected actin network (group) influenced by filament length and density^60^.

### Long-time PINE (Experiment: emergent dynamics SYNC)

We applied the model together with PINE to investigate the emergent process of cell division^66^. In order to investigate nanoscopic dynamics, we benchmarked the temporal capabilities of PINE against fluorescence super-resolution (Fig. 4b). Benchmarking nanoprobe-labeled actin bundles resolved by PINE with fluorophore-labeled actin bundles resolved by fluorescence super-resolution, subdiffraction resolution was preserved under continuous analysis using PINE, whereas with fluorescence super-resolution, subdiffraction capabilities were lost over time (<200 s) with continuous exposure to illumination. To validate long-time observation capabilities, we followed the temporal evolution of cellular architectures (actin) (Fig. 4c) and observed nanoscale rearrangements below the diffraction limit (Fig. S22) in agreement with theoretical modeling (Figs. S23, S24; Supplemental Note III). PINE can uniquely reveal individual constituents at the sub-10 nm scale, their meso- and macroscale group level coordination, simultaneously with the temporal emergence of collective processes over time (Fig. 1b iii).

If macroscale movements and shape changes are linked to the individual constituents, we reasoned that individuals and groups should exhibit coordinated behavior. To test this hypothesis, we followed individual, meso- and macro-scale reorganization as a parental cell grew and separated into daughter cells (Fig. 4d). To identify progression through cell division, we assessed variations in nuclear features^67^ over time (Fig. 4d i). Using PINE, we observed a macroscale expansion-contraction behavior (Fig. S26) consistent with literature^68^, where the cell area of parental cells initially expanded corresponding to G_1_, S, and G_2_ phases (corresponding to decreased connectivity in the model); thereafter, cell area contracted corresponding to M phase (corresponding to increased connectivity in the model), and then expanded as parental cells divided into daughter cells (corresponding to decreased connectivity in the model). Shape changes are known to be related to the cytoskeleton^69^; however, how individual constituents contribute to macroscale reorganization remain incompletely understood. Using PINE, we observed the sub-10 nm width of individual filaments remained consistent over time, indicating actin maintained as individual filaments (Fig. 4d iii). By following hundreds of individual constituents (904 filaments), we discovered individual filaments also underwent expansion-contraction behavior at the individual level (Fig. S27) synchronized with macroscale shape changes: (i) length of individual filaments initially contracted during G_1_, S, and G_2_ phases (corresponding to decreased connectivity in the model). (ii) next, length of individual filaments expanded during the M phase (corresponding to increased connectivity in the model). (iii) finally, the length of individual filaments contracted as parental cells divided into daughter cells (corresponding to decreased connectivity in the model). At the mesoscale, the density of individual filaments also exhibited expansion-contraction behavior observed by PINE (Fig. S28) coordinated with macroscale shape changes. During G_1_, S, and G_2_ phases, the density of individual filaments decreased (corresponding to decreased connectivity in the model). In the M phase, the density of individual filaments increased (corresponding to increased connectivity in the model). Finally, the density of individual filaments decreased as parental cells divided into daughter cells (corresponding to decreased connectivity in the model). No expansion-contraction behavior was observed in the undivided control (Fig. 4e ii). Taken together, PINE revealed emergent dynamics in which individuals and groups exhibited synchronized reorganization at the individual, meso- and macro-scale levels (Fig. 4e ii).

## Discussion

In discussion, the method introduced here can be used to overcome time limitations of fluorescence super-resolution for long-time nanoscopy. Using nonbleaching PINE, we demonstrated dynamic nanoscopy of living matter over 250 h, more than two orders of magnitude outperforming state-of-the-art fluorescence super-resolution. We resolved sub-10 nm cellular architectures and followed the emergence of their coordinated reorganization during the cell division process. We introduced a model based on local interactions of individuals to describe how individual constituents can synchronize with group level movements and shape changes at the macroscale. Presented here, we applied the model to the cell division process. This simple model provides a direct link between individual constituents and overall shape changes at the macroscale. The mechanism of coordination we proposed here in the model showed local interactions between neighboring individual constituents are sufficient to emerge into group level reorganization (Fig. 4e). Analogous to disruption of animal herds under attack^70^, how individual-group coordinated reorganization may be disrupted in disease are possibilities to be explored in the future. This is important to our understanding of dynamical emergent processes involved in cellular reorganization, self-organization, and pattern formation. This model can be applied to other soft matter, such as shape changing materials^71^.

In this work, we achieved PINE-resolved images with nanoprobe densities of ~10^2^/µm^2^, whereas higher nanoprobe densities are typically utilized in traditional fluorescence super-resolution. Since the distribution of distances from every nanoprobe to its first neighbor follows a distribution function^50^ (Fig. S19), underlying protein architectures can be identified for nanoprobe labeling densities of ~10^2^/µm^2^, albeit lower than nanoprobe densities typically used in traditional fluorescence super-resolution. This offers an advantage for minimizing excessive nanoprobe labeling which may affect observed dynamics. As with traditional fluorescence super-resolution, PINE also depends on labeling with nanoprobes. Underlying protein architectures which are not labeled cannot be identified. PINE is based on statistics. We demonstrated labeling densities of ~10^2^/µm^2^ were sufficient to identify underlying architectures based on nanoprobe distributions. However, if labeling densities are insufficiently low, it is possible distributions may be misidentified as random although underlying architectures may be present.

PINE has the potential for in vivo nanoscopy. A current limitation is the nanoprobe size for sufficient scattering. In the future, in vivo sub-10 nm nanoprobes displaying geometric singularities for high field generation (Fig. S29) can be designed to be modulated by phase-intensity to overcome this limitation with interferometry. PINE has the potential for four-dimensional (4-D) nanoscopy (*t, x, y, z*). A current limitation is the sample depth and background scattering. In the future, volume (sample depth) can be achieved by employing PINE with light-sheets (i.e., optical z sectioning) and background subtraction algorithms. This could lead to exciting studies of long timescale processes, such as emergent processes, evolutionary processes, ageing, and age-related phenomena. New control methods, such as subdiffraction optical tweezers, used in conjunction with PINE, would create exciting possibilities for spatiotemporal control of in vivo processes. In conclusion, we believe PINE will open new nanoscopic opportunities for investigations demanding long-time observation windows.

## Methods

### PI Device Fabrication

PI was fabricated by a deposition process. To prepare the precursor, 4-(3-acryloyoxypropyloxy) benzoic acid 2-methyl-1,4-phenylene ester 15 wt% (*n*_*o*_ = 1.508, *n*_*e*_ = 1.687), 1-hydroxycyclohexyl phenyl ketone 4 wt% and 4-cyano-4′-pentylbiphenyl (*n*_*o*_ = 1.707, *n*_*e*_ = 1.534) 81 wt% were mixed. Substrates were rubbed for alignment, hydroxylated by immersing in piranha (3:1 sulfuric acid: hydrogen peroxide) for 30 s, rinsed in isopropanol and deionized water three times, and dried. At room temperature, precursor was deposited (30 µm thickness) and cured (365 nm ultraviolet irradiation at 20 mW/cm^2^ for 5 min) between two polyethylene terephthalate-indium tin oxide substrates (127 µm thickness) on top of  a polyvinyl alcohol-iodine layer (180 µm thickness). Polyvinyl alcohol (180 µm thickness) was layered on top of the aforementioned layers. To characterize material alignment, crossed polarization with a polarizer in the illumination path and an analyzer in the detection path was set up on a transmission mode brightfield microscope. Polarization angle was then varied from 0° to 45°. To characterize response time, optical path consisted of a beam generated using a 660 nm laser incident on PI. A power meter was used to measure the output light intensity. Applied voltage to control PI was generated using a function generator.

### Experimental setup

Darkfield (Olympus) (Fig. 1c, S30)—configured with white light source (transmission mode), tunable laser (reflection mode), wet and dry condensers (1.2–1.4 numerical aperture), ×40, ×60, and ×100 objectives, PI, flow chamber, heating elements to keep the stage and flow chamber constant at 37 °C, CO_2_ incubator connected to the flow chamber, peristaltic perfusion pump connected to the flow chamber, piezostage xyz with z-adaptive correction on top of micrometer stage (Madcity), cameras: low noise CCD (Hamamatsu), highspeed CMOS (Excelitas)—was set up on an isolation platform on top of an anti-vibration table (Newport). A flow chamber containing a transparent viewing region was built for flow of reagents and outfitted with a multi-syringe pump. Voltage signals were sent from LabView software (National Instruments) on a computer through a Daq card (National Instruments) and a connector block (National Instruments) to PI. The connector block, at the same time, sent the trigger signals to CCD to externally trigger the image acquisitions. During image acquisition, images were acquired over the modulation range *γ* = 0−2π rad (34 increments) within one second.

### Imaging

#### PINE_1_

The innovation is long-time sub-10 nm nanoscopy to follow emergent nanoscale-to-macroscale reorganization. Deconvolution algorithm^2^ was used to generate high-resolution images from images acquired over the modulation range in MATLAB (MathWorks). To locate nanoprobes, the average of the scattering signal volume power and the noise volume power was used as a threshold to identify nanoprobes, where $I(x,y)$ was identified as a peak if the amplitude of $I(x,y)$ was larger than any surroundings. The peaks’ positions detected was $({Pk}{x}_{i},{Pk}{y}_{i})$ with $i$ representing the *i*-th peak:

8 $$X({Pkx},{Pky})=	\,\mathop{{{{{{\rm{argmin}}}}}}}\limits_{{X}_{1}}\{{{||}h*{X}_{1}+{iDCT}\left({X}_{2}\right)}\\ 	-{Y\cdot {{{{{\rm{ln}}}}}}\left(h*{X}_{1}+{iDCT}\left({X}_{2}\right)\right){||}}_{1}+{\lambda }_{1}{{||}{X}_{1}{||}}_{1}+{\lambda }_{2}{{||}{X}_{2}{||}}_{1}\}$$

where *h* is the point spread function, *iDCT* is the inverse discrete cosine transform, *Y* is the diffraction-limited image, $X({Pk}{x}_{i},{Pk}{y}_{i})$ is the localization, *X*_1_ is the deconvolved image, *X*_2_ is the background noise and *λ*_1_, *λ*_2_ are the regularization parameters.

#### PINE_2_

To obtain sub-10 nm information, the distribution of precisely localized nanoprobes formed a pattern of the underlying protein architecture denoted as

9 $${X}_{i}=\left({x}_{i},{y}_{i},{z}_{i}\right)\in {{{{{{\mathcal{R}}}}}}}^{3}$$

From the distribution of precisely localized nanoprobes, a two-dimensional surface in three-dimensional space is denoted by a vector function $S:{{{{{{\mathcal{R}}}}}}}^{2}\to {{{{{{\mathcal{R}}}}}}}^{3}$:

10 $${{{{{\bf{S}}}}}}\left({p}_{1},{p}_{2}\right)=\left\{\begin{array}{c}{S}_{x}({p}_{1},{p}_{2})\\ {S}_{y}({p}_{1},{p}_{2})\\ {S}_{z}({p}_{1},{p}_{2})\end{array}\right.$$

where $({p}_{1},{p}_{2})$ is the position on the surface under its natural coordinates. From the distribution of peak positions of nanoprobes, surface or curvilinear features ${f}:{{{{{{\mathcal{R}}}}}}}^{1}\to {{{{{{\mathcal{R}}}}}}}^{2}$ is extracted with the regression:

11 $${{{{{\bf{f}}}}}}\left(p\right)=\left\{\begin{array}{c}{f}_{x}(p)\\ {f}_{y}(p)\end{array}\right.$$

where *p* is the position on the curve under its natural coordinates. The projection index is defined as ${{{{{{\mathcal{P}}}}}}}_{f}:{{{{{{\mathcal{R}}}}}}}^{2}\to {{{{{{\mathcal{R}}}}}}}^{1}$:

12 $$E\left(\right.X{{|}}{{{{{{\mathcal{P}}}}}}}_{f}\left(X\right)={{{{{\bf{f}}}}}}\left(p\right)$$

Then the distribution of the distance parameter *χ*(*p*) is the function of the position: $\chi \left(p\right)=\frac{1}{n}{\left|\left|\left({Pk}{x}_{i},{Pk}{y}_{i}\right)-{{{{{\bf{f}}}}}}\left(p\right)\right|\right|}_{2}$ (Eq. 4) where $n$ is the number of nanoprobes in the nearest region of ${{{{{\bf{f}}}}}}\left(p\right),$ and $\left({{Pkx}}_{i},{{Pky}}_{i}\right)=X$ satisfies Eq. 12. Equation 4 minimizes the Euclidean distance from nanoprobe positions to their projection ${{{{{\bf{f}}}}}}(p)$. Set of images at different focal planes were captured. At each focal plane, images were acquired over the modulation range and processed using a deconvolution algorithm  to obtain deconvoluted high-resolution images. From the high-resolution images, area, width, length, and density were analyzed in Fiji.

### PI device model simulation

The multilayer film consisted of a polyvinyl alcohol layer as a fixed retarding element for reshaping, 4′-Pentyl-4-biphenylcarbonitrile nematic liquid crystalline layer as a variable retarding element for phase modulation, and polyvinyl alcohol-iodine layer as a fixed linear polarizing element for phase-to-intensity conversion. The output electric field was calculated using Eq. 1 in MATLAB (MathWorks). The electric field amplitudes and directions were determined by assigning a time-dependent component ($E\left(t\right)={real}\left(E\right){{cos }}\left({wt}\right)+{imag}\left(E\right){{sin }}\left({wt}\right)$) to the complex form of electric field to trace the electric field over 2π. The amplitudes of intensity before entering the device and after exiting the device were calculated by Eq. 2 using the square root of sum of electric field amplitude squares ($I=\,\sqrt{{E}_{x}^{2}+{E}_{y}^{2}}$).

### SYNC model simulation

Group sizes consisted of *N* = 50–1000 individuals, and initial conditions were connectivity *c* = 0.1–10.0, short-range space *ρ* = 70–700 nm, and long-range space *Ρ* = 5 μm consistent with experiments. Individuals were randomly positioned within a short-range space following Eq. 5 in MATLAB (MathWorks). Time step was Δ*t* for each frame with a total time of 600Δ*t*. For each individual, its neighbors within the long-range space and short-range space were determined. The direction the individual moved was then calculated using Eq. 7, where its movement vector was the product of the direction vector and the step size. Output connection $\left(1-{{{{{\mathcal{C}}}}}}\right)$: To calculate ${{{{{\mathcal{C}}}}}}$, the ratio of change in the average distance between neighbors at time *t* and the theoretical maximum distance between neighbors was calculated. Since intuitively output connection is higher with smaller ${{{{{\mathcal{C}}}}}}$, output connection was defined as $\left(1-{{{{{\mathcal{C}}}}}}\right)$.

### Statistics and reproducibility

All representative images reflect a minimum of three replicates.

### Reporting summary

Further information on research design is available in the Nature Portfolio Reporting Summary linked to this article.

## Supplementary information

Supplementary Information

Reporting Summary

## Data Availability

Data are available within the article and Supplementary, or available from the corresponding author upon request.

## Code availability

Code are available within the Supplementary, or available from the corresponding author upon request.
