# Supplementary material for: Phase intensity nanoscope (PINE) opens long-time investigation windows of living matter
Source: Nat Commun. 2023 Jul 18;14:4318. doi: 10.1038/s41467-023-39624-w (PMC10354063; doi:10.1038/s41467-023-39624-w)
Supplement: Supplementary file 1 — Supplementary Information [file 41467_2023_39624_MOESM1_ESM.pdf]

Supporting information

Phase intensity nanoscope PINE nanoscopy opens long-time investigation windows of living matter

*Guangjie Cui[1#], Yunbo Liu[1#], Di Zu[1], Xintao Zhao[1], Zhijia Zhang[1], Do Young Kim[1], Pramith Senaratne[1], Aaron Fox[1], David Sept[2], Younggeun Park[3], Somin Eunice Lee[1]**

[1]Department of Electrical & Computer Engineering, Biomedical Engineering, Applied Physics, Biointerfaces Institute, Macromolecular Science & Engineering, University of Michigan, Ann Arbor, Michigan USA
[2]Department of Biomedical Engineering, University of Michigan, Ann Arbor, Michigan USA
[3]Department of Mechanical Engineering, University of Michigan, Ann Arbor, Michigan USA
[#] Authors equally contributed.
* To whom correspondence should be addressed. sleee@umich.edu

Materials

Chemical reagents

4-(3-acryloyoxypropyloxy) benzoic acid 2-methyl-1,4-phenylene ester (RM257), 1-Hydroxycyclo hexyl phenyl ketone (Irgacure 184), 4-cyano-4′ -pentylbiphenyl (5CB), polyethylene terephthalate-indium tin oxide (PET-ITO), hexadecyltrimethylammonium chloride (CTAC), hexadecyltrimethylammonium bromide (CTAB), sodium oleate (NaOL), hydrogen tetrachloroaurate (III) trihydrate ($HAuCl_4 \cdot 3H_2O$), acetone, calcium chloride ($CaCl_2$), 2-propanol (IPA), L-ascorbic acid (AA), potassium chloride (KCl), silver nitrate ($AgNO_3$), sodium borohydride ($NaBH_4$), hydrochloric acid (HCl), 6-mercaptohexanoic acid (MHA), 1-dodecanethiol (DDT), toluene, methanol, 2-mercaptoethanol, sodium phosphate dibasic (Na_2HPO_4), sodium chloride (NaCl) were purchased from Sigma-Aldrich. Polyvinyl alcohol (PVA), polyvinyl alcohol-iodine (PVA-I) were purchased from Techspec. Deionized (DI) water with resistivity 18.2 MΩ was used.

Biological reagents

Methoxy polyethylene glycol thiol (mPEG-SH), 4-(2-Hydroxyethyl)piperazine-1-ethanesulfonic acid (HEPES), poly(ethylene glycol) 2-mercaptoethyl ether acetic acid (SH-PEG-COOH), 2-(N-Morpholino)ethanesulfonic acid (MES) monohydrate, N-(3-Dimethylaminopropyl)-N'-ethylcarbodiimide hydrochloride (EDC), N-hydroxysulfosuccinimide sodium salt (NHS), adenosine 5'-triphosphate disodium salt hydrate (ATP), adenosine 5'-triphosphate disodium salt hydrate (ATP), Tris (2-varboxyethyl) phosphine HCl (TCEP-HCl Tris), ethylenediaminetetraacetic acid (EDTA), magnesium chloride hexahydrate ($MgCl_2 \cdot 6H_2O$), N-hydroxysulfosuccinimide sodium salt (Sulfo-NHS), Dulbecco's phosphate buffered saline (DPBS), G-actin protein, and methylcellulose were purchased from Sigma-Aldrich. Antibody recognizing actin was purchased from Santa Cruz. Fluorophore-labeled G-actin protein, bolvine serum albumin (BSA), 4%

paraformaldehyde (PFA), Bisbenzimide (Hoechst), 4′,6-diamidino-2-phenylindole (DAPI), Dulbecco's modified eagle medium (DMEM), trypsin- Ethylenediaminetetraacetic acid (EDTA), Phosphate buffered saline (PBS) and Fluoroshield Mounting Medium were purchased from Thermo Fisher Scientific. Human neuroblastoma cell line (SH-SY5Y) was purchased from the American Type Culture Collection (ATCC).

Sample Preparation

Nanoprobe Synthesis

In order for nanoscopic imaging, scaling up to distributions of nanoprobes as well as structurally stabilized nanoprobes are required. To achieve 100% CTA+ free necessary for structural stabilization, gold nanorods 59 nm × 20 nm were synthesized by a bromide-free seed-mediated growth followed by an adaptation of round-trip phase transfer to achieve CTA+ free and passivate surfaces with 90% SH-PEG-COOH and 10% mPEG-SH[1]. To crosslink, EDC/NHS bioconjugation chemistry was employed to form NH_2- groups on the nanoprobes. Activated nanoprobes (OD=15, 500 µL) and actin antibody (200 µg/mL) were crosslinked by mixing at a 1:1 ratio for 90 minutes while gentle rotating. After crosslinking, anti-actin-nanoprobes were washed 3 times by centrifugation. After the final wash, supernatant was decanted and anti-actin-nanoprobes were resuspended in 500 µL PBS. To verify CTA+ free, elemental compositions of samples were analyzed by X-ray photoelectron spectroscopy (XPS) analysis.

Fixed Cells

Cells were cultured in media supplemented with 10% FBS and maintained in a 37°C incubator with 5% CO_2 humidified air. Cells were cultured to 70% confluency on fibronectin-coated glass

coverslips. Blocking with 3% BSA in culture medium was then performed for 30 minutes followed by washing 3 times with PBS and incubated in culture medium. Cells were incubated with anti-actin-nanorods (OD=15, 75 μL) in culture medium for six hours followed by 3 times washing with PBS. Cells were treated with 4% paraformaldehyde and then incubated with DAPI for 10 minutes, followed by washing 3 times.

Live Cells

Cells were cultured in media supplemented with 10% FBS and maintained in a 37°C incubator with 5% CO_2 humidified air. Cells were cultured to 70% confluency on fibronectin-coated glass coverslips in culture medium with 10% FBS at 37°C. To synchronize cells, cells were incubated in culture medium with 0% FBS for 24 hours at 37°C. To re-enter the cell cycle, cells were incubated in culture medium with 20% FBS for 30 hours at 37°C and then returned back to culture medium with 10% FBS. Blocking with 3% BSA in culture medium was then performed for 30 minutes followed by washing 3 times with PBS and incubated in culture medium. Cells were incubated with anti-actin-nanorods (OD=15, 75 μL) in culture medium for six hours followed by washing 3 times with PBS. Cells on coverslips were then assembled into a chamber on a temperature-controlled microscope stage at 37°C. Culture medium was perfused into the chamber at 0.5 mL/min at 37°C with 5% CO_2. Cells were cultured and imaged for 2 days to follow dividing cells and cells which did not divide.

In Vitro Actin

Unlabeled G-actin from rabbit skeletal muscle (1 mg/mL) was prepared in storage buffer (5 mM Tris-HCl pH 8.0, 0.2 mM $CaCl_2$, 0.2 mM ATP, 0.5 mM TCEP). To crosslink, EDC/NHS bioconjugation chemistry was employed to form NH_2- groups on the nanoprobes. Activated

nanoprobes (OD=15, 100 μL) were added to the actin solution at a 1:1 ratio and incubated for 1 hr. After the crosslinking step, the sample was gently washed 3 times with DI water. For the fluorescent imaging, unlabeled G-actin was added to fluoroscein labeled G-actin at a 1:1 ratio in storage buffer (5 mM Tris-HCl pH 8.0, 0.2 mM $CaCl_2$, 0.2 mM ATP, 0.5 mM TCEP). Labeled actin was flowed into a chamber coated with 1% methylcellulose and imaged for 1.5 weeks.

XPS

To characterize material composition, samples were deposited on clean silicon substrates for X-ray photoelectron spectroscopy (XPS) analysis. Spectra were acquired using monochromatic Al X-rays at 14 kV and 12 mA, 1 eV scan step size, 60 s sweep time with analyzer at 160 eV pass energy, averaged by two times of sweeping to remove the noise, and calibrated with Au $4f_{7/2}$ at 84.00 eV.

Electron Beam Lithography

Gold nanorods 125 nm × 44 nm were fabricated using electron beam lithography on a glass substrate. A 100 nm thick 950k A2 PMMA photoresist was first spin-coated onto a glass slide, followed by Ni beads drop-cast on the edge of the substrate to aid focusing in the e-beam process. Gold nanorods were then patterned by electron beam lithography (Jeol 6300FS) using a beam size of 1.8 nm and a step size of 0.125 nm to ensure uniform patterning while preserving the designed shape. After developing the patterns, a layer of chromium (20 Å) and a layer of gold (300 Å) were deposited by ebeam evaporation (Enerjet). A lift-off process was subsequently performed using acetone to dissolve the photoresist.

SEM

Samples were blocked with 1.5% BSA in PBS for 30 minutes at room temperature before being rinsed three times with PBS. Materials were dehydrated by sequentially rinsing in 50, 60, 70, 80, 90, and 100 % ethanol for 30 minutes each time. Images were acquired (Hitachi SU-8000) after drying materials in the air for one day.

Code

```
%%
clear,clc

sample = 'sample'; %Sample number
psf_file = 'psf.tif';

%% Reading in files
Img_psf = double(imread(psf_file));
h = Img_psf/sum(Img_psf(:));
figure(1)
imshow(h,[0,max(h(:))]);
title('PSF');
nFrame = 34;

for m=1:nFrame
    % Read in raw images
    y_2d(:,:,m) = double(imread([sample,'\img\f',sprintf('%02d',m),'.tif']));
    figure(2)
    subplot(1,nFrame,m)
    imshow(y_2d(:,:,m),[0,max(max(y_2d(:,:,m)))]);
end

comb = zeros(size(y_2d(:,:,1)));
for m=1:nFrame
    comb = comb+y_2d(:,:,m)/nFrame;
    figure(3);
    imshow (comb,[0,max(comb(:))]);
    title(['Diffraction limited images ',sample]);
end
close all

%% Deconvolution
opts.lambda = [0.07,5];
opts.max_iter = 10000;
```

```
opts.tol = 1e-6;
opts.L0 = 1;
opts.eta = 1.1;

nFrame = size(y_2d,3); %Numbers of frame
X_deconv = zeros(size(y_2d));
B_deconv = zeros(size(y_2d));

fprintf('========Data for reconstruction Exported=========\n');
parfor m =1:nFrame

    z_m = double(y_2d(:,:,m));
    ini = zeros([size(z_m),2]); %Initial values
    avg = sum(z_m(:))/numel(z_m);
    ini(1,1,2) = avg*sqrt(numel(z_m));
    fprintf('Deconvolute frame %d, %d frames in total\n',m,nFrame);
    tic;[X_deconv(:,:,m),B_deconv(:,:,m),cost] = decon(z_m, h, ini, opts); toc;
end

%% Image Generation
x_recon1 = zeros(size(y_2d,1),size(y_2d,2));

for m=1: nFrame
    Recon_tmp = X_deconv(:,:,m);
    nFig_m = ceil(m/6);
    m_fig = mod(m,6);
    if m_fig ==0
        m_fig =6;
    end
    figure(nFig_m);
    subplot(2,3,m_fig);
    imshow(Recon_tmp,[]);
    title(['Image for frame ',num2str(m)]);
    x_recon1 = x_recon1 + double(Recon_tmp);
end

figure (nFig_m+1);
imshow(x_recon1,[0,max(x_recon1(:))]);
title('Reconstruction');

% Convert diffraction_limited image
raw_img = comb/max(comb(:));
raw_img = uint16(round(65535*raw_img));
rgb_raw = cat(3, raw_img, raw_img, raw_img);

% Convert reconstruction image
recon_img = x_recon1/max(x_recon1(:));
recon_img = uint16(round(65535*recon_img));
rgb_recon = cat(3, recon_img, zeros(size(recon_img)), zeros(size(recon_img)));

% Plot the overlap image
figure (nFig_m+2);
imshow(rgb_recon+rgb_raw/2);
title('Overlap image');
```

```
%% utilities
function res = norm1(X)
    res = sum(abs(X(:)));
end

function res = normE2(X)
    A = X.^2;
    res = sum(A(:));
end

function res = prox(X, lambda)
    x1 = X(:,:,1);
    x2 = X(:,:,2);
    lamb1 = lambda(1);
    lamb2 = lambda(2);

    res (:,:,1) = subplus((x1)-lamb1);
    res (:,:,2) = subplus(abs(x2)-lamb2).*sign(x2);

end

function [X1, X2, cost] = decon(Y, H, Xinit, opts)
%% Value of lambda
    lambda = opts.lambda;

    function res = calc_f(mu)
        res = sum(sum((mu - Y.*log(mu))));
    end

    function res = calc_F(mu,X)
        res = calc_f(mu)+lambda(1)*norm1(X(:,:,1))+lambda(2)*norm1(X(:,:,2));
    end

%% Gradient Function: grad(f(mu))
    function res = grad(mu)
        tmp = ones(size(Y))-Y./mu;
        res(:,:,1) = conv2(tmp,H,'same');
        res(:,:,2) = dct2(tmp);
    end

%% Q function: calculate the Q function
    function res = calc_Q(mu,x, y, L)
        x1 = x(:,:,1);
        y1 = y(:,:,1);
        x2 = x(:,:,2);
        y2 = y(:,:,2);
        det1 = x1-y1;
        det2 = x2-y2;

        grad_total = grad(mu);
        grad1 = grad_total(:,:,1);
        grad2 = grad_total(:,:,2);

        res = calc_f(mu)+det1(:)'*grad1(:)+det2(:)'*grad2(:)...
             +L/2*normE2(x-y)+lambda(1)*norm1(x1)+lambda(2)*norm1(x2);
```

```
     end
%% Deconvolution
    [X1, X2, cost] = decon_btrack(@calc_F, @grad, @calc_Q, H, Xinit, opts);
end

function [X1, X2, cost] = decon_btrack(calc_F, grad, calc_Q, H, Xinit, opts)
%% Initialization
    max_iter = opts.max_iter;
    lambda = opts.lambda;
    tol = opts.tol;
    x = Xinit;
    y = Xinit;
    t = 1;
    k = 0;
    L = opts.L0;
    eta = opts.eta;
    cost=zeros(1,max_iter);
    fprintf('********** Deconvolution Start ***********\n');
%% Loop
    tic;
    while  k < max_iter
        k = k + 1;
        L_bar = L;
    %% Find the L_bar (step size determination)
        mu_y = conv2(y(:,:,1),H,'same')+idct2(y(:,:,2));
        while true
            zk = prox(y - feval(grad, mu_y)/L_bar, lambda/L_bar);
            mu_zk = conv2(zk(:,:,1),H,'same')+idct2(zk(:,:,2));
            F = feval(calc_F, mu_zk, zk);
            Q = calc_Q(mu_y, zk, y, L_bar);
            if F <= Q
                break;
            end
            L_bar = L_bar*eta;
            L = L_bar;
        end

        x_new = zk;
        t_new = 0.5*(1 + sqrt(1 + 4*t^2));
        y_new = x_new + (t - 1)/t_new * (x_new - x);
        cost(k) = F;
    %% Early shutoff
        diff = norm1(x_new(:,:,1)-x(:,:,1))/norm1(x(:,:,1));
        if  diff < tol
            fprintf('converged at %dth iterations\n',k);
            break;
        end

    %% Update
        x = x_new;
        t = t_new;
        y = y_new;

    %% Show Progress
```

```
        if (mod(k,100)==0)
            time_k = toc;
            cost_k = cost(k);
            fprintf('iter = %d, convergence = %f,cost f = %f, time = %f s\n', k, diff,cost_k, time_k);
        end

    end
    X1 = x_new(:,:,1); % Deconvoluted images (reconstructed NR distribution)
    X2 = x_new(:,:,2); % Background & noise
    fprintf('********** Iteration End ***********\n');
end
```

Supplementary Note I: Validation of displacement-free PI

Considering a single gold nanorod as a model nonbleaching nanoprobe, when a moving polarizer/analyzer was used to angularly rotate the polarization from 0° to 180°, the Airy pattern of the imaged nanoprobe traced a circle at the imaging plane over the range of polarization[3]. Displacements during rotation can be corrected down to a limit imposed by the adjustment accuracy of the correcting elements. Spatial errors remain even with motorized polarizers/analyzers and/or optimized arrangements such as reducing the distance between the camera and the polarizer/analyzer.

In this work, we designed an integrated phase-intensity multilayer thin film (herein referred to as PI) to be displacement-free. Distinctly different from our previously reported work constructed with standard waveplate stacks[3], here the compact multilayer design was integrated into the infinity space between the objective and tube lens, enabling high transmission for nanoscopy. The optical axis of PI was stationary such that the center coordinates during modulation (x_γ, y_γ) were equal to the center coordinates prior to modulation (x_0, y_0), enabling zero displacement for precise nanoscopy (Eq. 3). For validation of displacement-free PI, the scattered electric field from a single nanorod was reshaped by PI as $\mathbf{E} = E_x e^{i\delta_x} + E_y e^{i\delta_y}$. Using PI, we

modulated γ from 0 to 2π which varies the phase difference between E_x and E_y from $-\pi$ το π according to Eq. 1. After phase to intensity conversion by PI, we observed the nanorod remained precisely at the same position in the imaging plane over the modulation range following Eq. 3 using PI (Figure S11). As a measure of displacement, we quantified the root mean square deviation of the images acquired over the modulation range. The normalized root-mean-square deviation of the images was calculated for each pixel, where the experimental value of the intensity was compared with the theoretical value of the intensity fitted to sinusoidal functions over the modulation range.

$$\text{RMSD}\ (m,n) = \frac{1}{I_{e,\max}(m,n) - I_{e,\min}(m,n)}\sqrt{\frac{\sum_{i=1}^{N}(I_{e,i}(m,n) - I_{s,i}(m,n))^2}{N}}, \tag{13}$$

where N is the number of frames, I_e is the experimental value of the intensity, I_s is the theoretical value fitted to a sinusoidal functions, and $I_{e,max}$ and $I_{e,min}$ are the maximum and minimum intensity at pixel (m, n) over all phase retardances. We observed near zero error, confirming PI was displacement-free limited by noise (Figure S12). Environmental noise can be suppressed by isolating vibration sources. Image noise can be suppressed with low noise and single photon CCDs. Zero displacement of PI eliminates potential imprecise localization from displacement artifacts, laying the foundation for precise localization of nanoprobe populations. To generate scatter plot (Figure 2b), images were repeatedly acquired over the modulation range γ from 0 to 2π, generating image stacks of ~500 total frames. From the image stacks, distinct populations corresponded to each distinct nanorod from which spatial positions were quantified. The spatial positions of each nanorod were determined by taking the median value.

Supplementary Note II: Scaling up to distributions of nanoprobes formed architecturally distinct patterns of underlying cellular architectures resolved by PINE

Actin was labeled with nanoprobes (gold nanorods) bound with antibodies targeting actin. PINE images showed details of architecturally distinct structures which were indistinguishable in the diffraction-limited images. Detailed structures of intersected bundles were distinguishable by PINE (Figure S17). Multiple line profiles along the PINE-resolved structure clearly showed that the intersected bundles initially converged (Figure S17a, i), then intersected, and finally diverged again (Figure S17a, ii and iii) which cannot be seen in the diffraction-limited image. Branched structures were also visible by PINE (Figure S17b). Line profiles along the PINE-resolved structure showed a daughter bundle (Figure S17b, i) protruded from the side of a mother bundle (Figure S17b, ii and iii), forming a Y-shaped branch that was undetectable in the diffraction-limited image. Finally, higher ordered structures were revealed by PINE that were not visible in the diffraction-limited images. Parallel, intersected and branched structures combined to form complex networks (Figure S17c).

Supplementary Note III: Validation of temporal capabilities

We validated the response time of the PI to determine the timescale of dynamic activities that can be captured. To validate long timescale temporal capabilities, we followed the temporal evolution of cellular architectures (actin) over time scales of weeks (Figure 4c). We observed local rearrangements below the diffraction limit (Figure S22) in agreement with theoretical modeling (Figure S23). No local rearrangements in the negative control (Figure S24).

For the theoretical model, we solved the mass transport equations for diluted species $\nabla \cdot \mathbf{J_i} = R_i$ and $\mathbf{J_i} = -D_i \nabla c_i$ where $\mathbf{J_i}$ is the mass flux (mol/(m^2·s)), R_i is the reaction rate for the species and D_i is the diffusion coefficient (m^2/s) to estimate the diffusion of molecules around actin

structures with a finite element analysis method (FEA, COMSOL Multiphysics software). The geometries were 1,800 nm in length and 100 nm in width with different ξ:15°, 30°, 45°, 60°, 75°, 90°. The aqueous environment around the actin structures was assumed to be 50 mM KCl and 2 mM $MgCl_2$. The material properties, dynamic viscosity and temperature, were set to 0.899 Pa·s and 293 K, respectively[4]. Triangle-type mesh was applied to the entire domain, with a minimum element size of 0.1 nm and a maximum element size of 100 nm. The boundary condition for the media domain was set at a constant concentration of 0.2 mM. The diffusion coefficient within the media and the consumption rate was assumed to be 3.54×10^{-10} m^2/s and 40 μmol/L/s, respectively[5–7].

By theoretically modeling the concentration of molecules in the nanoenvironment of actin nanostructures, we observed low concentration profiles of molecules as low as ~10^{-8} mM occurred in structurally confined sites, and high concentration profiles of molecules in unconfined sites. We systematically investigated the structural parameter ξ from 15° to 90° on the concentration of molecules in the nanoenvironment of actin nanostructures (Figure S23). We selected three representative sites: A (unconfined site), B: (confined site), and C (highly confined site) and observed the concentration of molecules was correlated with ξ.

Supplementary Figures

Method	Space: super-resolution	Time: observation window	Reference
STED	0.14λ	30 min	[8]
	0.08λ	20 sec	[9]
	0.14λ	26 min	[10]
	0.09λ	150 sec	[11,12]
	0.27λ	60 sec	[13,14]
	0.19λ	60 sec	[14,15]
GSD	0.04λ	10 min	[16]
	0.08λ	2.5 sec	[17]
STORM	0.04λ	8 min	[18]
	0.08λ	30 sec	[19]
	0.2λ	50 sec	[20]
PALM	0.11λ	25 min	[21]
	0.07λ	50 sec	[22]
	0.17λ	8 min	[23]
	0.08λ	132 sec	[24]
**PINE**	**0.01λ - 0.14λ**	**40 ms -1.5 weeks**	**Fig. 3, 4, S5**

Figure S1. State-of-the-art comparison of Space: super-resolution vs. Time: observation window parameters.

Reference	Number of resolved nanoprobes
[25]	9
[26]	6
[27]	4
[28]	12
[29]	3
Fig. 2, S6-S8	2-4
Fig. 3	2,860
Fig. S16	7,769

Figure S2. State-of-the-art comparison of number of resolved nanoprobes.

Material	Thickness
Polyvinyl alcohol	180 µm
Polyethylene terephthalate	127 µm x 2 = 254 µm
Indium tin oxide	1.5 nm x 2 = 3.0 nm
4-cyano-4′-pentylbiphenyl and 4-(3-acryloyoxypropyloxy) benzoic acid 2-methyl-1,4-phenylene ester	30 µm
Polyvinyl alcohol-iodine	180 µm

Figure S3. Material and thickness of each layer in PI.

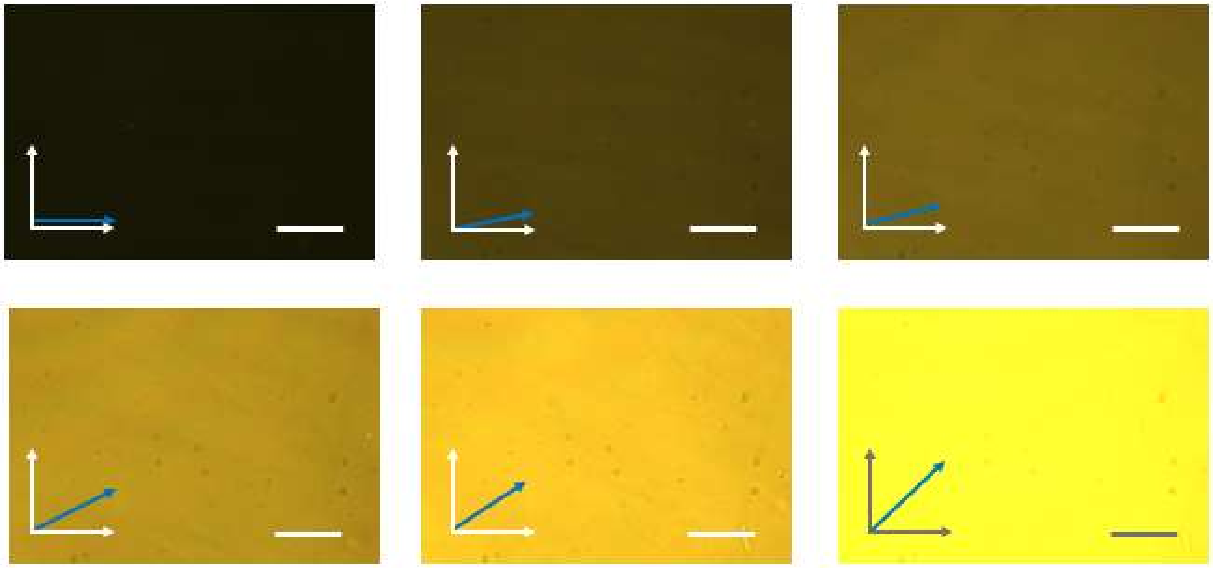

Figure S4. Material alignment characterization. 4-(3-acryloyoxypropyloxy) benzoic acid 2-methyl-1,4-phenylene ester 15 wt%, 1-hydroxycyclohexyl phenyl ketone 4 wt%, 4-cyano-4′ -pentylbiphenyl 81 wt% was deposited and cured at room temperature under ultraviolet irradiation at 20 mW/cm^2 for 5 minutes. Sample was analyzed under crossed polarization in transmission mode bright field microscopy. Polarization angle was varied from 0° to 45°. We observed homogenous alignment over the entire sample. Scale bar: 50 μm.

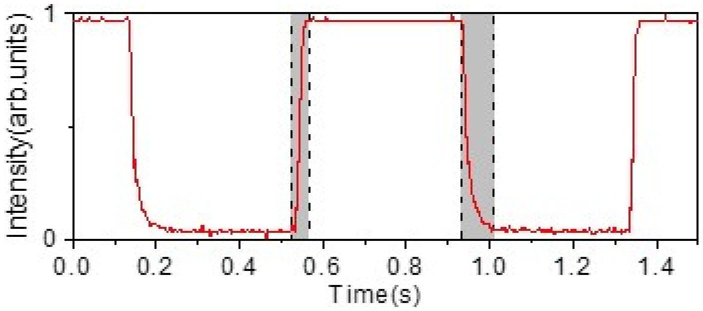

Figure S5. Millisecond response time of PI. To characterize response time, optical path consisted of a beam generated using a 660nm laser incident on PI. Power meter was used to measure the output light intensity. Applied voltage to control PI was generated using a function generator. Graph of response time: Intensity over time.

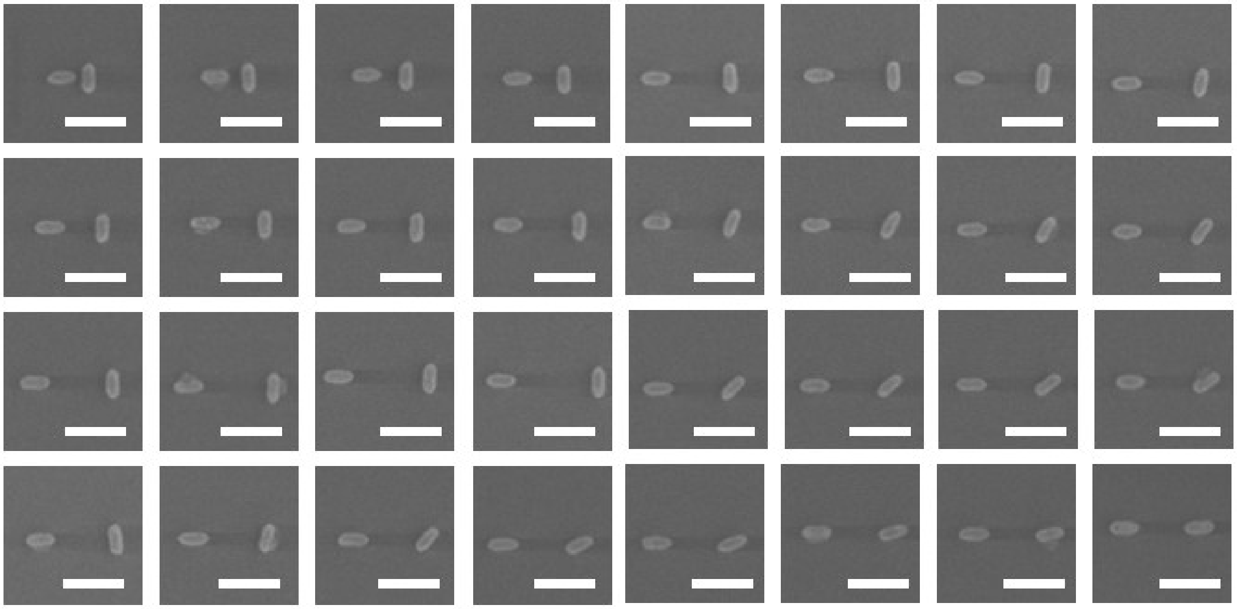

Figure S6. Multiple nanorods in a diffraction limited region corresponding to Figure 2. Gold nanorods were fabricated using electron beam lithography on a glass substrate. Scale bar: 250 nm.

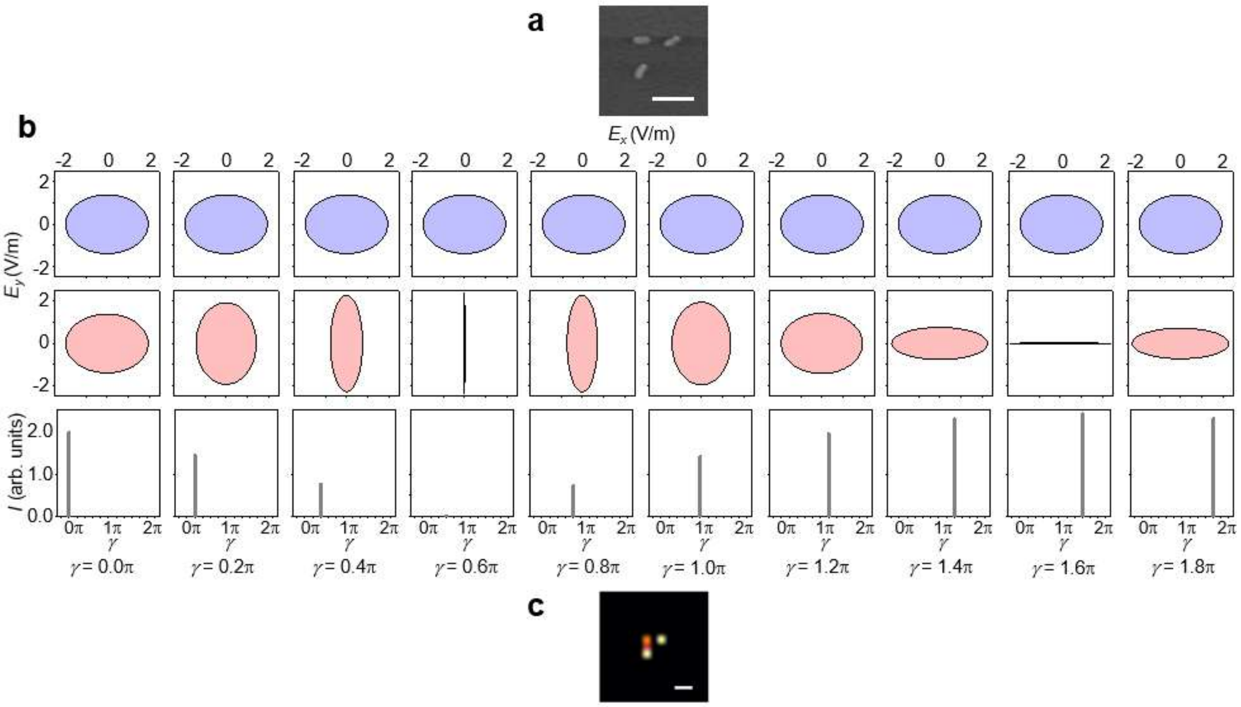

Figure S7. Multiple nanorods in a diffraction-limited region. (a) Scanning electron microscopy image of multiple gold nanorods in a diffraction-limited region fabricated by electron beam lithography on a glass substrate. Scale bar: 250 nm. **(b)** Phase-intensity PI: Calculated electric field and intensity amplitudes where scattered light was reshaped (top row), phase modulated (middle row), and intensity modulated (bottom row). **(c)** PINE-resolved image of multiple nanorods in a diffraction-limited region. Color represents intensity after phase-to-intensity conversion. Scale bar: 200 nm.

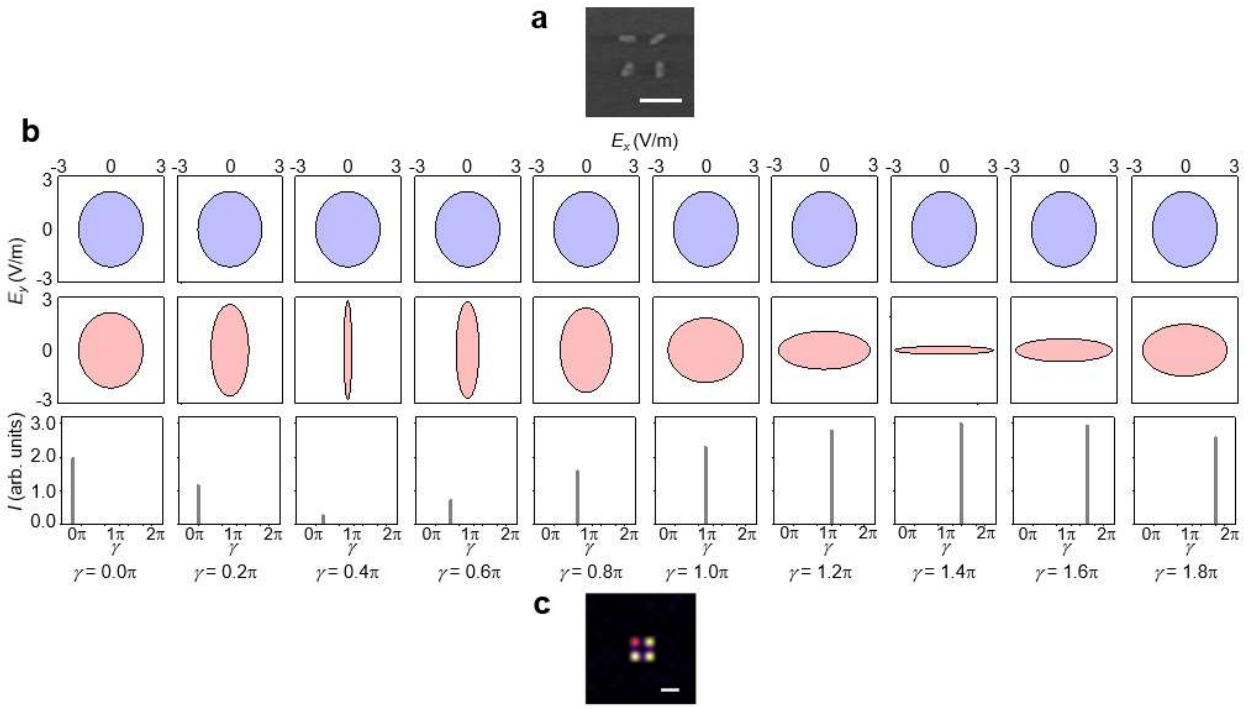

Figure S8. Multiple nanorods in a diffraction-limited region. (a) Scanning electron microscopy image of multiple gold nanorods in a diffraction-limited region fabricated by electron beam lithography on a glass substrate. Scale bar: 250 nm. **(b)** Phase-intensity PI: Calculated electric field and intensity amplitudes where scattered light was reshaped (top row), phase modulated (middle row), and intensity modulated (bottom row). **(c)** PINE-resolved image of multiple nanorods in a diffraction-limited region. Color represents intensity after phase-to-intensity conversion. Scale bar: 200 nm.

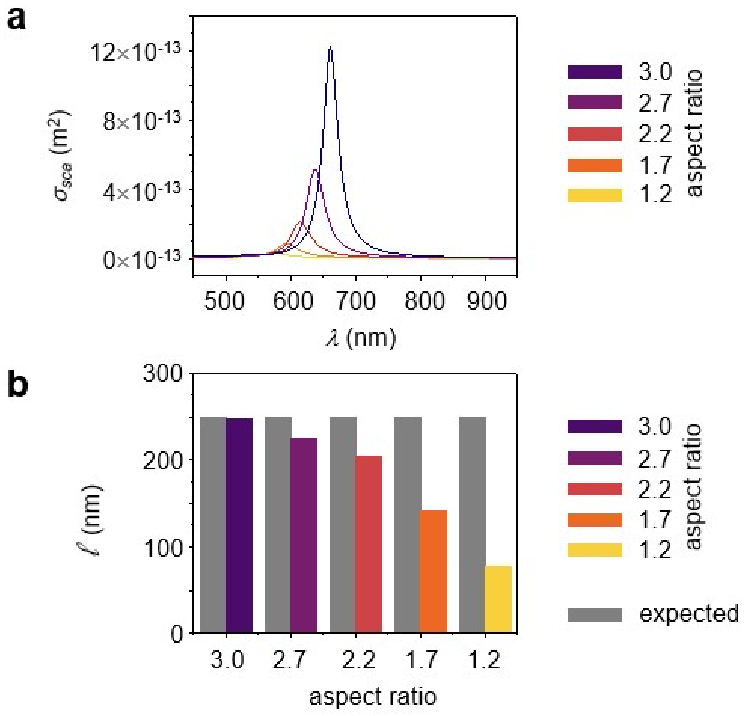

Figure S9. Localization optimized based on aspect ratio. Simulation of proximal gold nanorods where the geometrical parameter α was set to 30° and expected subdiffraction ℓ was set to 250 nm which was below the diffraction limit of 450 nm. **(a)** Scattering cross-section versus wavelength for various aspect ratio gold nanorods using Mie-Gans model. The extinction ratio of a gold nanorod was estimated by taking the ratio between the longitudinal peak and transverse peak intensity. **(b)** Extinction ratio was then used as the ground truth image intensities for simulation of proximal gold nanorods. At aspect ratio 3.0, ℓ obtained by localization (dark purple bar) matched to the expected ℓ (grey bar), indicating precise localization. As the aspect ratio decreased to 1.2, ℓ obtained by localization (yellow bar) no longer matched to the expected ℓ (grey bar), indicating imprecise localization. Legend: grey color: expected ℓ, dark purple color: 3.0 aspect ratio (30 nm × 90 nm), purple color: 2.7 aspect ratio (30 nm × 80 nm), dark orange color:

2.2 aspect ratio (30 nm × 65 nm), orange color: 1.7 aspect ratio (30 nm × 50 nm), yellow color: 1.2 aspect ratio (30 nm × 35 nm).

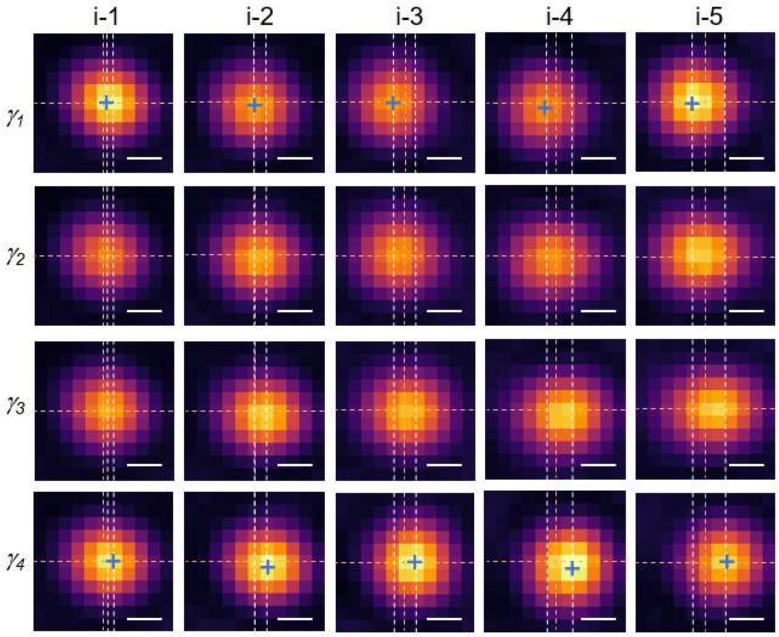

Figure S10. Representative darkfield images corresponding to Figure 2d. PI modulated γ from 0 to 2π during image acquisition. i-1 through i-5 correspond to Figure 2d. Scale bar: 400 nm.

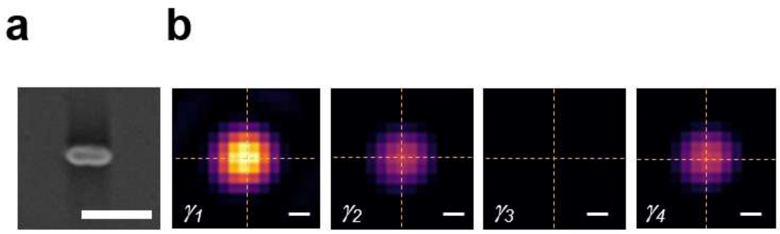

Figure S11. Experimental validation of displacement-free PI. (a) Scanning electron microscopy image of a single gold nanorod fabricated by electron beam lithography on a glass substrate. Scale bar: 240 nm. **(b)** Representative darkfield images acquired by PI at $\gamma_1 = 0.2\pi$, $\gamma_2 = 0.6\pi$, $\gamma_3 = 1.0\pi$, $\gamma_4 = 1.4\pi$. Scale bar: 200 nm.

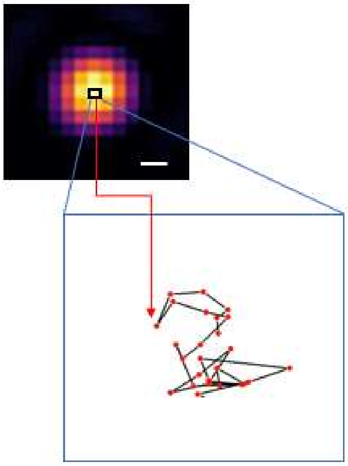

Figure S12. Environmental noise suppression. Representative darkfield image of single gold nanorod fabricated by electron beam lithography on a glass substrate. Inset: magnified view of random trajectories due to noise. Environmental noise can be suppressed by isolating vibration sources. Image noise can be suppressed with low noise and single photon CCDs. Scale bar: 200 nm.

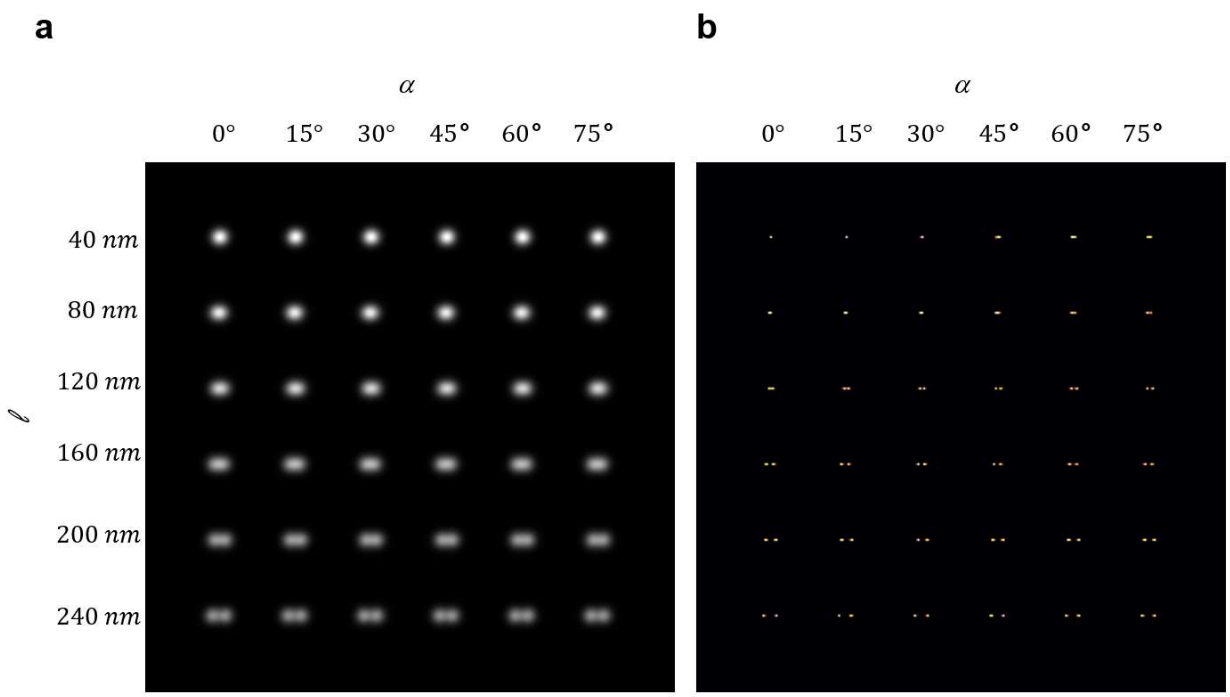

Figure S13. Localization. (a) Diffraction limited image of two proximal nanoprobes (nanorods) varying geometrical parameters α and ℓ. **(b)** PINE-resolved image of two proximal nanoprobes (nanorods) varying geometrical parameters α and ℓ.

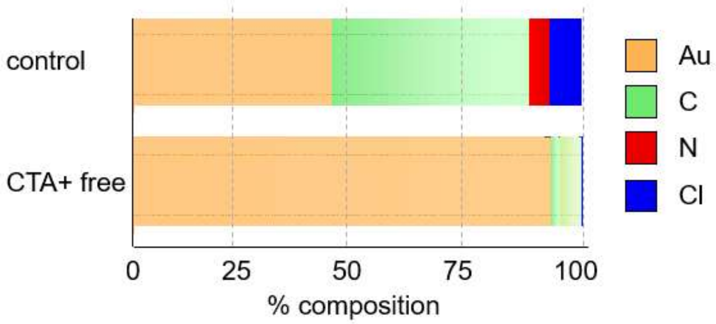

Figure S14. Quantitative elemental composition verifies 100% CTA+ free. Gold nanorods were synthesized by a bromide-free seed-mediated growth followed by an adaptation of round-trip phase transfer to achieve CTA+ free. To validate 100% CTA+ free, we analyzed the elemental concentration of N 1s originating from CTAC by X-ray photoelectron spectroscopy (XPS). Control shows strong N 1s at 399 eV. Absence of N 1s originating from CTAC verifies 100% CTA+ free[1]. (reproduced with permission of John Wiley and Sons, copyright 2020 Wiley-VCH GmbH)

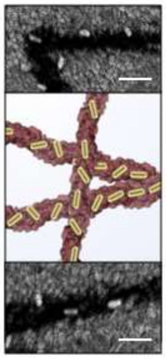

Figure S15. Scanning electron microscopy verifies nanoprobes randomly distributed along actin bundles. Scale bar: 150 nm.

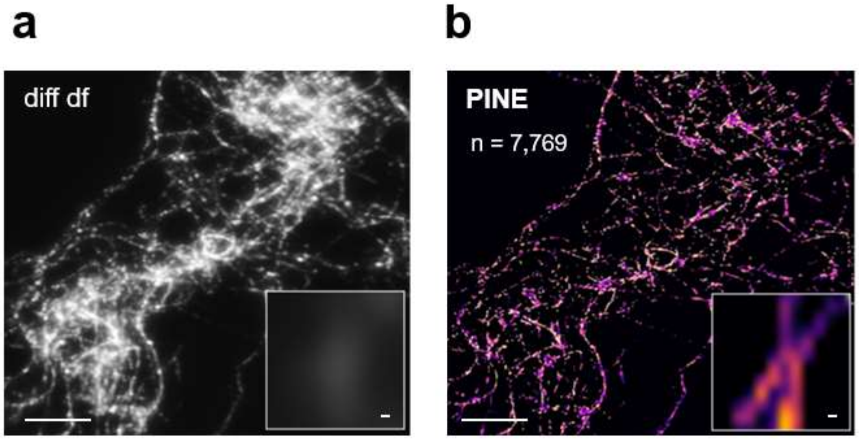

Figure S16. PINE scalably resolves population of 7, 769 nanoprobes labeling cellular architectures (actin). To locate nanoprobes, the average of the scattering signal volume power and the noise volume power was used as a threshold to identify nanoprobes, where $I(x, y)$ was identified as a peak if the amplitude of $I(x, y)$ was larger than any surroundings. Scale bar 10 μm. Inset: magnified PINE-resolved image of intersected cellular architectures (actin). Scale bar: 150 nm.

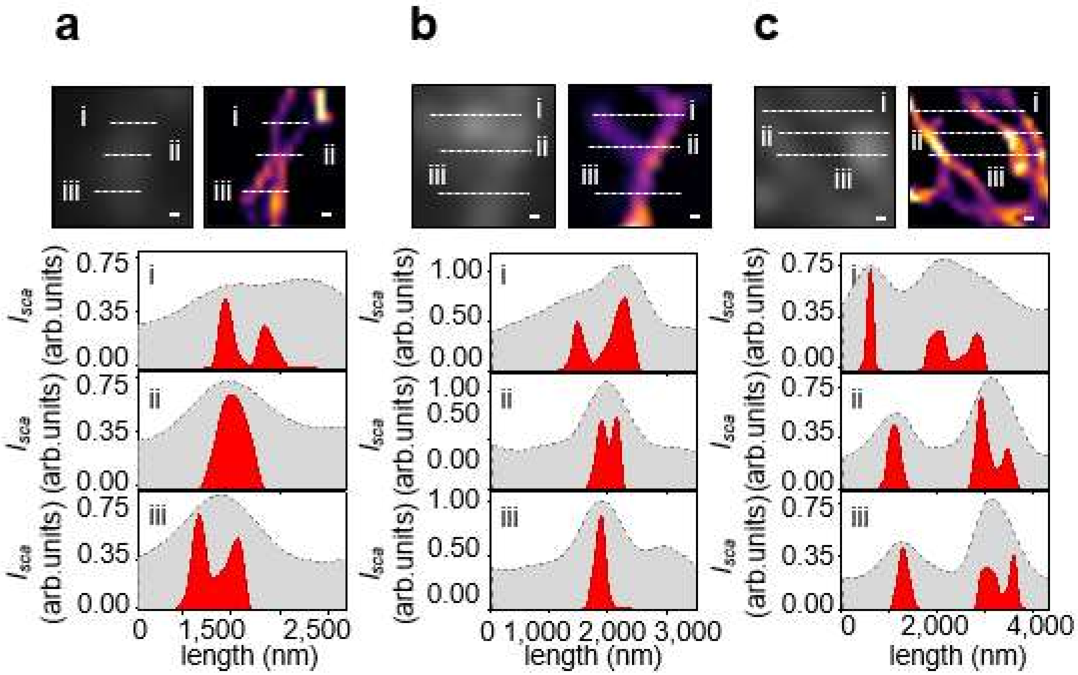

Figure S17. Scaling up to distributions of nanoprobes formed architecturally distinct patterns of underlying cellular architectures (actin) resolved by PINE. (a) Intersected actin nanostructure: diffraction-limited darkfield image. PINE-resolved intersected actin nanostructure. i, ii, iii are line profiles corresponding to profile plots of scattering intensity versus position where red curve: PINE-resolved, grey curve: diffraction-limited. **(b)** Branched actin nanostructure: diffraction-limited image. PINE-resolved branched actin nanostructure. i, ii, , iii are line profiles corresponding to profile plots of scattering intensity versus position along length where red curve: PINE-resolved, grey curve: diffraction-limited. **(c)** Complex actin nanostructure: diffraction-limited image. PINE-resolved complex actin nanostructure. i, ii, iii are line profiles corresponding to profile plots of scattering intensity versus position where red curve: PINE-resolved, grey curve: diffraction-limited. Scale bar: 150 nm.

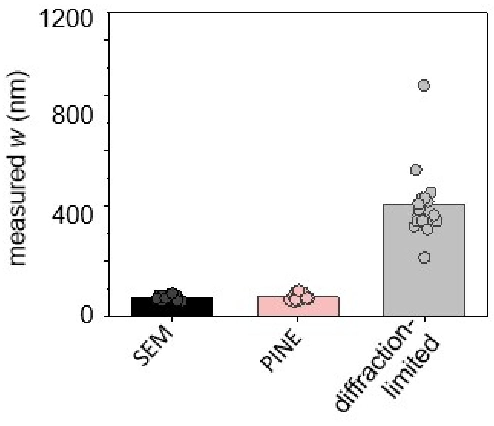

Figure S18. Benchmarking of PINE. Comparison of actin bundle widths resolved by PINE (pink) in agreement with measurements by scanning electron microscopy (black) in Figure S17. Diffraction-limited darkfield (grey). n = 16 replicates.

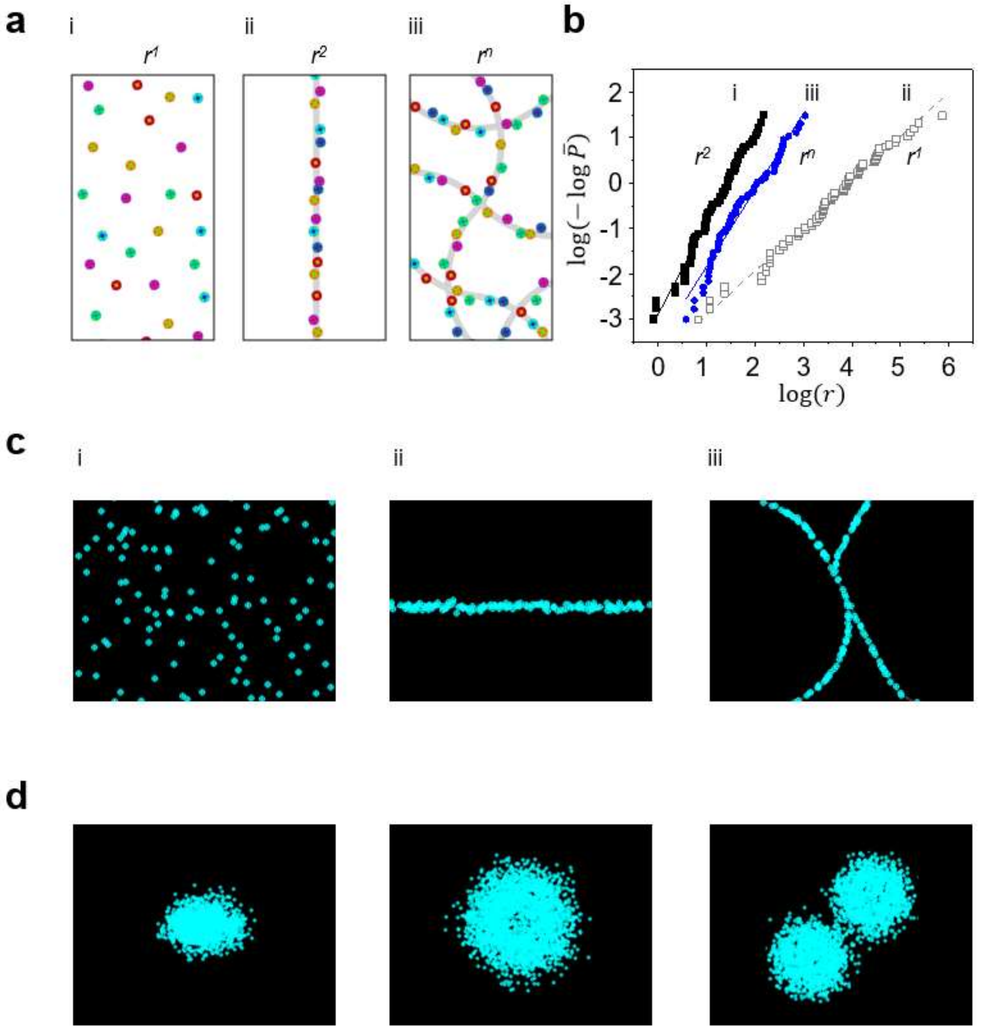

Figure S19. Architectures were identified based on distances to first neighbors. Simulations were conducted at nanoprobe labeling densities used in experiments. **(a)** Conceptual schematic of **i)** randomly distributed nanoprobes, **ii)** linear architecture labeled with nanoprobes, **iii)** complex architecture labeled with nanoprobes. **(b)** Graph of complementary cumulative distribution function $\bar{P}$ versus distance to first neighbor r. $\bar{P}$: probability of finding a neighbor greater than r. Nanoprobe distributions scale as r^n , where $1 \leq n \leq 2$. **i)** randomly distributed nanoprobes: r^1, **ii)** linear architecture labeled nanoprobes: r^2, **iii)** complex architecture labeled with nanoprobes: r^n . **(c) i)** Simulation of randomly distributed nanoprobes. **ii)** Simulation of linear architecture labeled

with nanoprobes, **iii)** Simulation of complex architecture labeled with nanoprobes. **(d)** Simulation of arbitrary architectures labeled with nanoprobes.

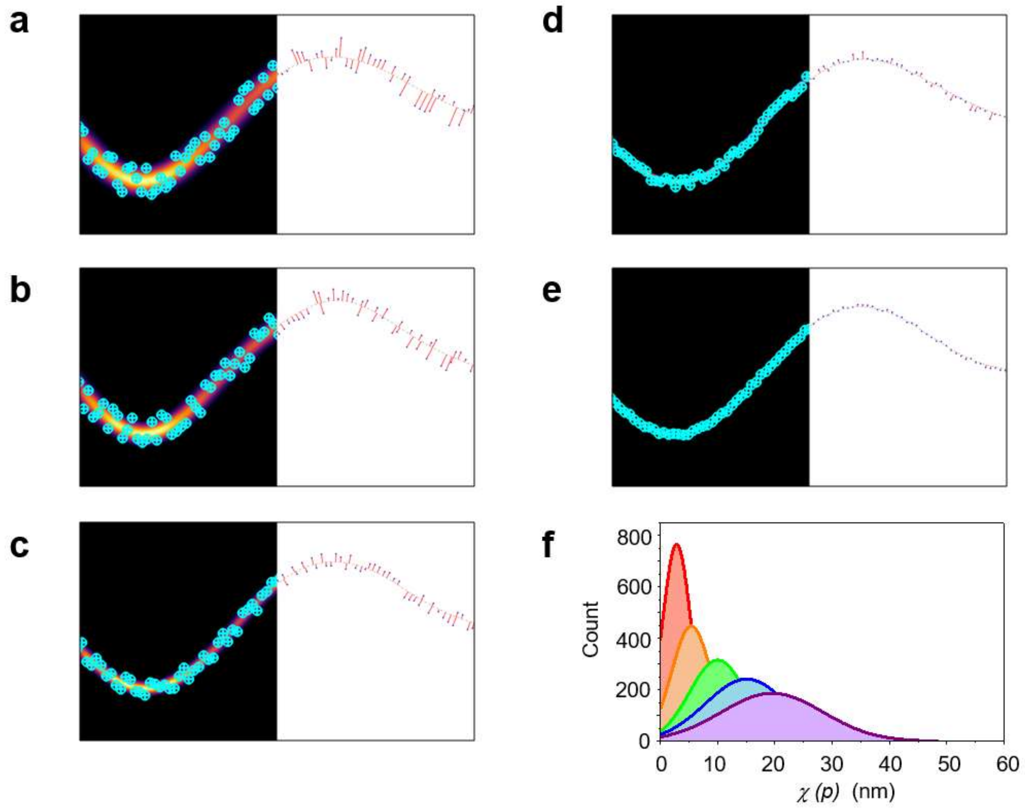

Figure S20. Variation of *χ(p)*. Left side represents precisely localized nanoprobe positions (Pkx_i, Pky_i). Right side represents $\boldsymbol{f}(p)$, the projection of nanoprobe positions. Simulation of: **(a)** *χ(p)* = 20 nm, **(b)** *χ(p)* = 15 nm, **(c)** *χ(p)* = 10 nm, **(d)** *χ(p)* = 5 nm, **(e)** *χ(p)* = 2 nm, **(f)** Histogram of average *χ(p)* from ten simulations corresponding to parts a, b, c, d, e: purple color *χ(p)* = 20 nm, blue color *χ(p)* = 15 nm, green color *χ(p)* = 10 nm, orange color: *χ(p)* = 5 nm, red color *χ(p)* = 2 nm.

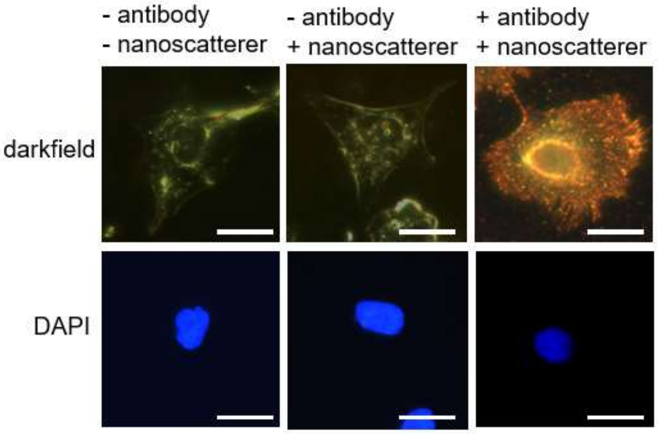

Figure S21. Specific labeling was confirmed via negative controls for nonspecificity of nanoprobe-antibody labeling of SH-SY5Y neuroblastoma cells. (left) Control: Darkfield and DAPI images of cells in the absence of nanoprobes and antibody. (middle) Control: Darkfield and DAPI images of cells in the presence of nanoprobes and absence of antibody. (right) Darkfield and DAPI images of cells in the presence of nanoprobes conjugated with antibody. Scale bar: 20 μm.

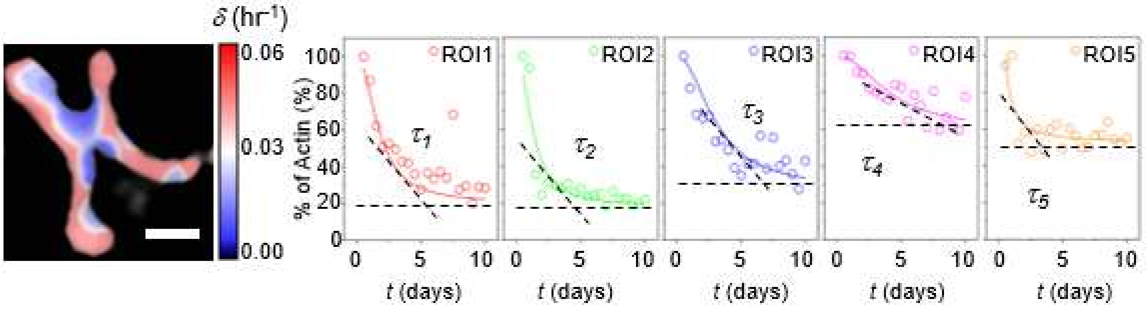

Figure S22. Nanoscopic temporal map of actin degradation rates δ constructed from stack of time course nanoscopy images in Figure 4c. Scale bar: 690 nm. Graphs of actin percentages over time measured in various nanoscale regions with heterogeneous decay constants: ROI1 τ_1 = 0.86 days^{-1}, ROI2 τ_2 = 1.44 days^{-1}, ROI3 τ_3 = 1.80 days^{-1}, ROI4 τ_4 = 4.00 days^{-1}, ROI5 τ_5 = 0.75 days^{-1}.

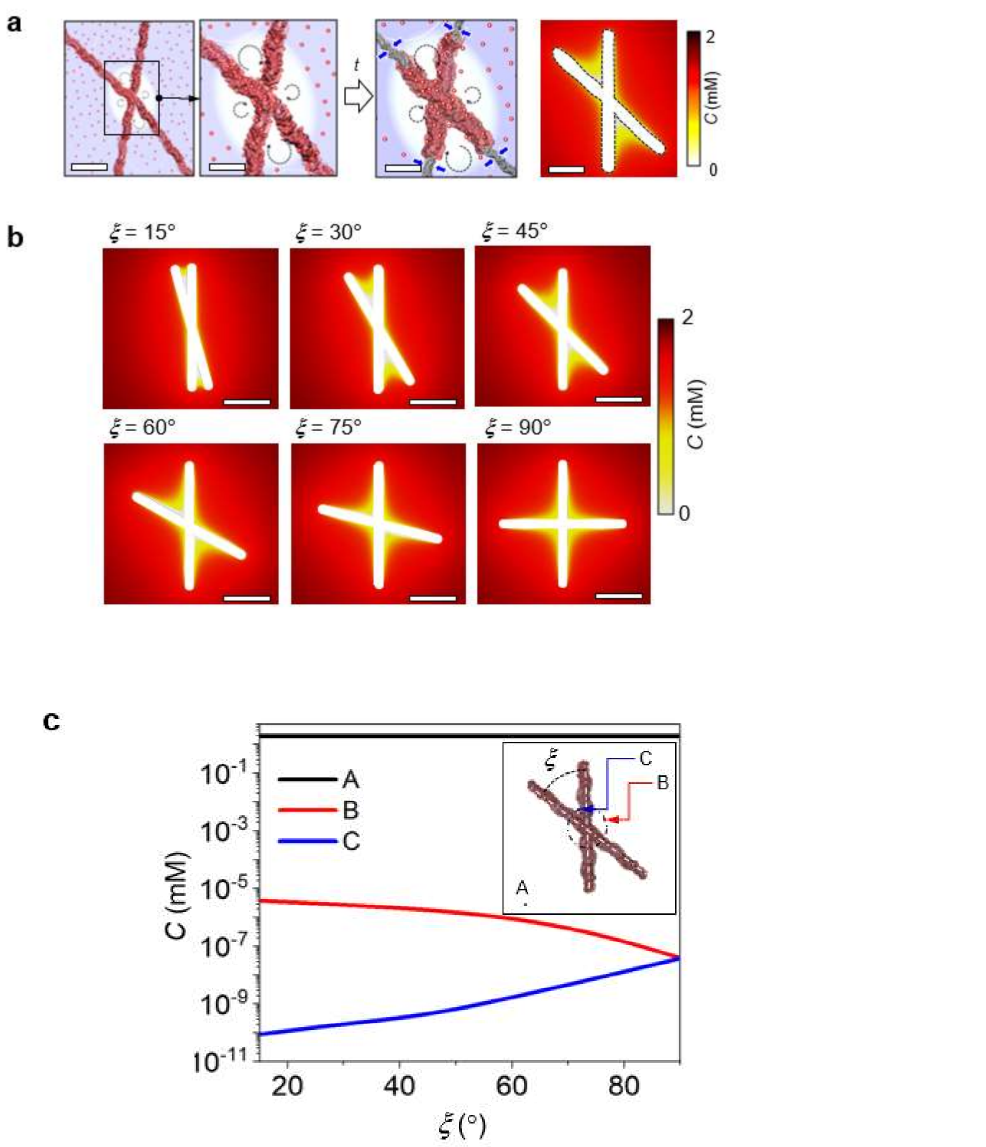

Figure S23. Geometry determines nanoscale structural confinement of actin structures. (a) Conceptual schematic of nanoscale structural confinement of molecules around an actin structure. **(b)** Concentration of molecules in the nanoenvironment of actin structures with different structural parameter ξ (scale bars = 500 nm) and **(c)** C at three different locations; A, B, and C as a function of ξ.

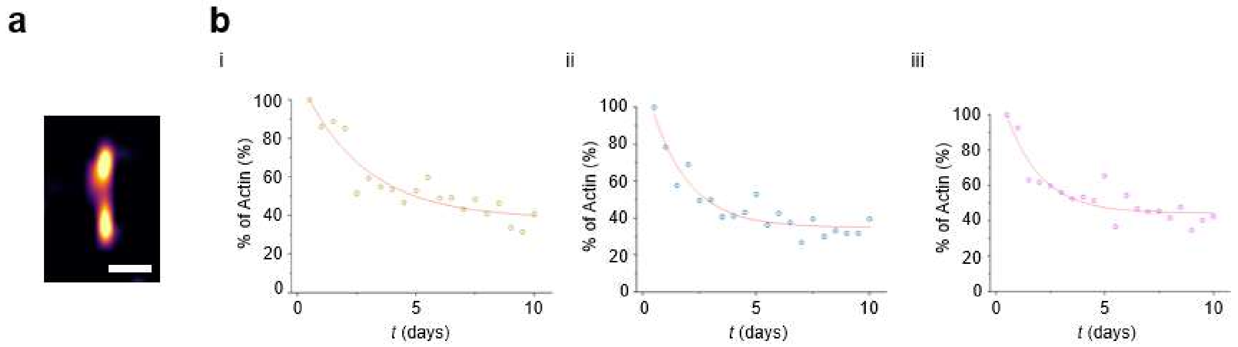

Figure S24. Negative control of temporally homogeneous degradation rates. (a) PINE resolved image. Scale bar: 690 nm, (b) i. Graphs of actin percentages over time measured in various nanoscale regions: i. ROI1, ii. ROI2, iii. ROI3.

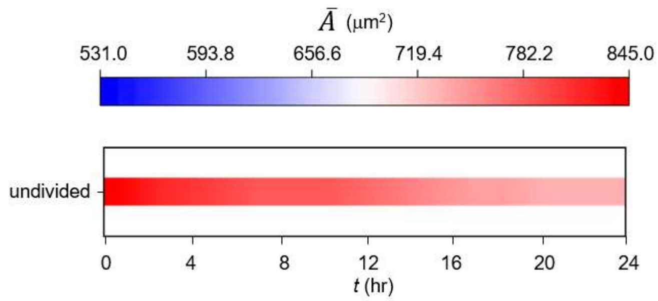

Figure S25. Undivided Control. Cells which did not divide were followed over time to calculate cell area over time. For comparison with experiment, scale bar was set to the same percentage change as the experiment. No expansion-contraction was observed over time. Parameter in Figure 4e: Mean cell area $\bar{A}$.

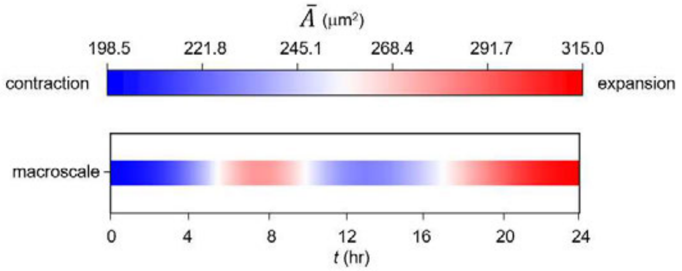

Figure S26. Cell division (macroscale). Contraction-expansion graph over time. Parameter in Figure 4e: Mean cell area $\bar{A}$.

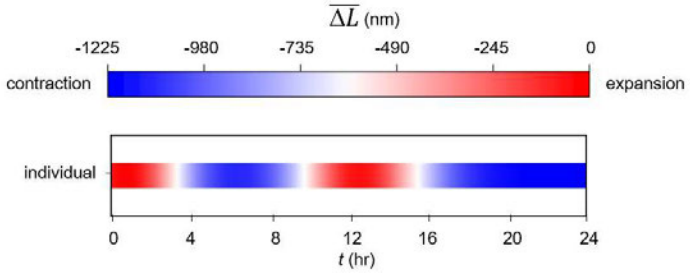

Figure S27. Cell division (individual). Contraction-expansion graph over time. Parameter in Figure 4e: Mean change in length of individual filaments $\overline{\Delta L}$.

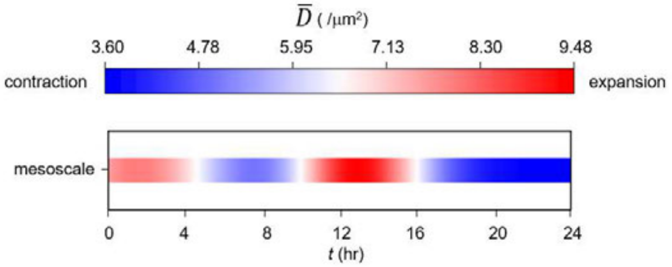

Figure S28. Cell division (mesoscale). Contraction-expansion graph over time. Parameter in Figure 4e: Mean density of individual filaments $\overline{D}$.

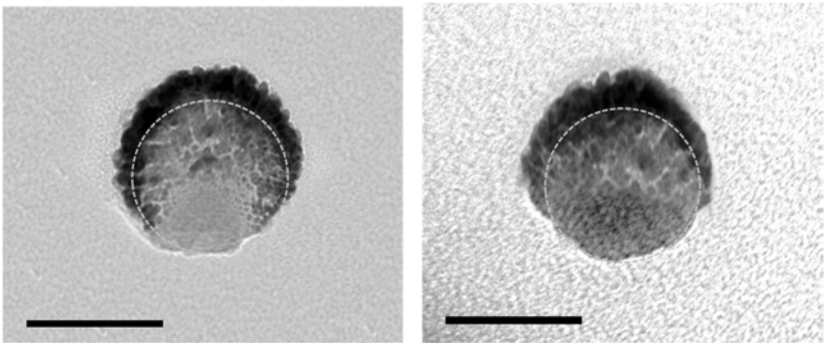

Figure S29. Multimaterial nanoscatterers. Sub-10 nm nanoscatterers can be obtained by tuning the geometrical parameters during the fabrication process. Mesoporous silica (12 nm) was arrayed on a SiO_2 substrate. Gold nanolayer was then formed by angle deposition and nanoscatterers were then harvested from the SiO_2 substrate. Scale bar: 20 nm.

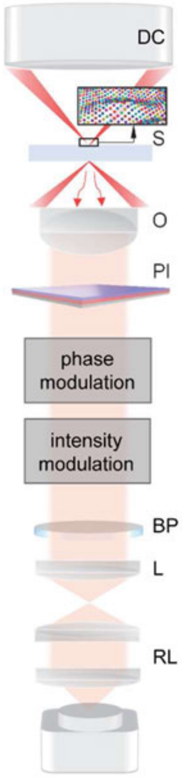

Figure S30. Set-up. Darkfield configuration: Darkfield condenser (DC) illuminates a nanoprobe-labeled live cell sample (S) in a temperature- and gas- controlled flow chamber. The collected scattered light by objective (O) is phase-intensity modulated (PI) and bandpass filtered (BP). To increase the system's magnification, relay lenses (RL) were added to increase the effective focal length of the tube lens (L). After phase-intensity separation, the resulting intensity variation corresponds to subsets of nanoprobes.

Supplementary References

1. Lin, W. K. *et al.* Optically and structurally stabilized plasmo-bio interlinking networks. *Adv. Mater. Interfaces* **2001370**, 1–9 (2020).

2. Beck, A. & Teboulle, M. A fast iterative shrinkage-thresholding algorithm. *Soc. Ind. Appl. Math. J. Imaging Sci.* **2**, 183–202 (2009).

3. Liu, Y., Zhang, Z., Park, Y. & Lee, S. E. Ultraprecision Imaging and Manipulation of Plasmonic Nanostructures by Integrated Nanoscopic Correction. *Small* **17**, 1–8 (2021).

4. Kestin, J., Khalifa, H. E. & Correia, R. J. Tables of the dynamic and kinematic viscosity of aqueous NaCl solutions in the temperature range 20-150°C and the pressure range 0.1-35 MPa. *J. Phys. Chem. Ref. Data* **10**, 71–88 (1981).

5. Hubley, M. J., Moerland, T. S. & Rosanske, R. C. Diffusion coefficients of atp and creatine phosphate in isolated muscle: pulsed gradient 31p nmr of small biological samples. *NMR Biomed.* **8**, 72–78 (1995).

6. Footer, M. J., Kerssemakers, J. W. J., Theriot, J. A. & Dogterom, M. Direct measurement of force generation by actin filament polymerization using an optical trap. *Proc. Natl. Acad. Sci. U. S. A.* **104**, 2181–2186 (2007).

7. Bernstein, B. W. & Bamburg, J. R. Actin-ATP hydrolysis is a major energy drain for neurons. *J. Neurosci.* **23**, 1–6 (2003).

8. Berning, S., Willig, K. I., Steffens, H., Dibaj, P. & Hell, S. W. Nanoscopy in a living mouse brain. *Science* **335**, 551 (2012).

9. Moneron, G. *et al.* Fast STED microscopy with continuous wave fiber lasers. *Opt. Express* **18**, 1302 (2010).

10. Urban, N. T., Willig, K. I., Hell, S. W. & Nägerl, U. V. STED nanoscopy of actin dynamics in synapses deep inside living brain slices. *Biophys. J.* **101**, 1277–1284 (2011).

11. Willig, K. I., Rizzoli, S. O., Westphal, V., Jahn, R. & Hell, S. W. STED microscopy reveals that synaptotagmin remains clustered after synaptic vesicle exocytosis. *Nature* **440**, 935–939 (2006).

12. König, I. *et al.* Single-molecule spectroscopy of protein conformational dynamics in live eukaryotic cells. *Nat. Methods* **12**, 773–779 (2015).

13. Klar, T. A. & Hell, S. W. Subdiffraction resolution in far-field fluorescence microscopy. *Opt. Lett.* **24**, 954 (1999).

14. Geddes, C. D., Cao, H. & Lakowicz, J. R. Enhanced photostability of ICG in close proximity to gold colloids. *Spectrochim Acta A Mol Biomol Spectrosc* **59**, 2611–2617 (2003).

15. Klar, T. A., Jakobs, S., Dyba, M., Egner, A. & Hell, S. W. Fluorescence microscopy with diffraction resolution barrier broken by stimulated emission. *Proc. Natl. Acad. Sci. U. S. A.* **97**, 8206–8210 (2000).

16. Dixon, R. E., Vivas, O., Hannigan, K. I. & Dickson, E. J. Ground state depletion super-resolution imaging in mammalian cells. *J. Vis. Exp.* **2017**, 1–9 (2017).

17. Fölling, J. *et al.* Fluorescence nanoscopy by ground-state depletion and single-molecule return. *Nat. Methods* **5**, 943–945 (2008).

18. Rust, M. J., Bates, M. & Zhuang, X. Sub-diffraction-limit imaging by stochastic optical reconstruction microscopy (STORM). *Nat. Methods* **3**, 793–795 (2006).

19. Shim, S. H. *et al.* Super-resolution fluorescence imaging of organelles in live cells with photoswitchable membrane probes. *Proc. Natl. Acad. Sci. U. S. A.* **109**, 13978–13983

(2012).

20. Bálint, Š., Vilanova, I. V., Álvarez, Á. S. & Lakadamyali, M. Correlative live-cell and superresolution microscopy reveals cargo transport dynamics at microtubule intersections. *Proc. Natl. Acad. Sci. U. S. A.* **110**, 3375–3380 (2013).

21. Shroff, H., Galbraith, C. G., Galbraith, J. A. & Betzig, E. Live-cell photoactivated localization microscopy of nanoscale adhesion dynamics. *Nat. Methods* **5**, 417–423 (2008).

22. Tatavarty, V., Kim, E. J., Rodionov, V. & Yu, J. Investigating sub-spine actin dynamics in rat hippocampal neurons with super-resolution optical imaging. *PLoS One* **4**, (2009).

23. Cisse, I. I. *et al.* Polymerase II Clustering in. *Science* **245**, 664–667 (2013).

24. Cox, S. *et al.* Bayesian localization microscopy reveals nanoscale podosome dynamics. *Nat. Methods* **9**, 195–200 (2012).

25. Ahn, S., Zhang, P., Yu, H., Lee, S. & Kang, S. H. Ultrasensitive detection of α-fetoprotein by total internal reflection scattering-based super-resolution microscopy for superlocalization of nano-immunoplasmonics. *Anal. Chem.* **88**, 11070–11076 (2016).

26. Cheng, X., Dai, D., Xu, D., He, Y. & Yeung, E. S. Subdiffraction-limited plasmonic imaging with anisotropic metal nanoparticles. *Anal. Chem.* **86**, 2303–2307 (2014).

27. Chakkarapani, S. K., Sun, Y., Lee, S., Fang, N. & Kang, S. H. Three-dimensional orientation of anisotropic plasmonic aggregates at intracellular nuclear indentation sites by integrated light sheet super-resolution microscopy. *ACS Nano* **12**, 4156–4163 (2018).

28. Wang, G., Sun, W., Luo, Y. & Fang, N. Resolving rotational motions of nano-objects in engineered environments and live cells with gold nanorods and differential interference contrast microscopy. *J. Am. Chem. Soc.* **132**, 16417–16422 (2010).

29. Zhang, P., Lee, S., Yu, H., Fang, N. & Kang, S. H. Super-resolution of fluorescence-free plasmonic nanoparticles using enhanced dark-field illumination based on wavelength-modulation. *Sci. Rep.* **5**, 1–9 (2015).
